# Expression and activation of nuclear hormone receptors result in neuronal differentiation and favorable prognosis in neuroblastoma

**DOI:** 10.1186/s13046-022-02399-x

**Published:** 2022-07-19

**Authors:** Lourdes Sainero-Alcolado, Muhammad Mushtaq, Judit Liaño-Pons, Aida Rodriguez-Garcia, Ye Yuan, Tong Liu, María Victoria Ruiz-Pérez, Susanne Schlisio, Oscar Bedoya-Reina, Marie Arsenian-Henriksson

**Affiliations:** 1 Department of Microbiology, Tumor and Cell Biology (MTC), Karolinska Institutet, SE-171 65 Stockholm, Sweden; 2 Present address: Department of Biotechnology, Faculty of Life Sciences and Informatics, Balochistan University of Information Technology, Engineering and Management Sciences, Quetta, 87300 Pakistan; 3 Present address: Department of Medicine, Center for Molecular Medicine (CMM), Karolinska Institutet, SE-171 64 Stockholm, Sweden

**Keywords:** Neuroblastoma, Nuclear hormone receptors, Neuronal differentiation, Metabolic reprogramming, Glucocorticoid receptor, Estrogen receptor α, Retinoic acid receptor α

## Abstract

**Background:**

Neuroblastoma (NB), a childhood tumor derived from the sympathetic nervous system, presents with heterogeneous clinical behavior. While some tumors regress spontaneously without medical intervention, others are resistant to therapy, associated with an aggressive phenotype. *MYCN*-amplification, frequently occurring in high-risk NB, is correlated with an undifferentiated phenotype and poor prognosis. Differentiation induction has been proposed as a therapeutic approach for high-risk NB. We have previously shown that MYCN maintains an undifferentiated state via regulation of the *miR-17 ~ 92* microRNA cluster, repressing the nuclear hormone receptors (NHRs) estrogen receptor alpha (ERα) and the glucocorticoid receptor (GR).

**Methods:**

Cell viability was determined by WST-1. Expression of differentiation markers was analyzed by Western blot, RT-qPCR, and immunofluorescence analysis. Metabolic phenotypes were studied using Agilent Extracellular Flux Analyzer, and accumulation of lipid droplets by Nile Red staining. Expression of angiogenesis, proliferation, and neuronal differentiation markers, and tumor sections were assessed by immunohistochemistry. Gene expression from NB patient as well as adrenal gland cohorts were analyzed using GraphPad Prism software (v.8) and GSEA (v4.0.3), while pseudo-time progression on post-natal adrenal gland cells from single-nuclei transcriptome data was computed using scVelo.

**Results:**

Here, we show that simultaneous activation of GR and ERα potentiated induction of neuronal differentiation, reduced NB cell viability in vitro, and decreased tumor burden in vivo. This was accompanied by a metabolic reprogramming manifested by changes in the glycolytic and mitochondrial functions and in lipid droplet accumulation. Activation of the retinoic acid receptor alpha (RARα) with all*-trans* retinoic acid (ATRA) further enhanced the differentiated phenotype as well as the metabolic switch. Single-cell nuclei transcriptome analysis of human adrenal glands indicated a sequential expression of *ERα*, *GR*, and *RARα* during development from progenitor to differentiated chromaffin cells. Further, in silico analysis revealed that patients with higher combined expression of *GR*, *ERα*, and *RARα* mRNA levels had elevated expression of neuronal differentiation markers and a favorable outcome.

**Conclusion:**

Together, our findings suggest that combination therapy involving activation of several NHRs could be a promising pharmacological approach for differentiation treatment of NB patients.

**Supplementary Information:**

The online version contains supplementary material available at 10.1186/s13046-022-02399-x.

## Background

Neuroblastoma (NB), an embryonal tumor originating from neural crest derivatives of the sympathetic nervous system, is the most common extracranial solid malignancy diagnosed in children during the first year of life [1, 2]. It presents a diverse clinical pattern ranging from spontaneous regression to widespread incurable disease. According to the International Neuroblastoma Staging System (INSS), NB is divided in five stages based on the severity and age of onset [1–3]. Despite intense multimodal treatment, ~ 40% of high-risk patients are incurable [4, 5]. Considerable efforts have been devoted to identifying drugs that induce differentiation as therapeutic options [6–8]. Retinoic acid (RA), a vitamin A derivative, is used as a differentiation and anti-neoplastic agent in cancer, including leukemia and several solid tumors [9]. The effect of RA is mediated by binding to the RA receptors (RARs), members of the nuclear hormone receptor (NHR) family. Once activated, RARs bind to specific RA response elements, triggering a downstream signalling cascade that results in differentiation [10]. In NB, this process is preceded by downregulation of MYCN and upregulation of nerve growth factor (NGF) receptors [11]. Neuroblastoma cells showed increased RA receptor α (RARα) levels in response to RA [12]. Importantly, 13-*cis*-RA is used as maintenance therapy in combination with immunotherapy in some European countries and in the United States for high-risk NB patients, however without stratification for *RARα* expression levels [13–15].

*MYCN-*amplification occurs in approximately 20% of all cases, and in around 40% of the high-risk NB group, linked with an undifferentiated phenotype and poor outcome [1, 2]. We have previously shown that MYCN promotes upregulation of the *miR-17 ~ 92* microRNA cluster, which in turn represses a plethora of target genes including the NHRs *estrogen receptor α* (*ERα*) and the *glucocorticoid receptor* (*GR*) [16–18]. The NHRs are unique therapeutic targets because they can be modulated with small molecules that mimic their natural ligands, enabling finetuning of their biological activities [19].

Glucocorticoid receptor activation via glucocorticoids results in anti-inflammatory, anti-proliferative, pro-apoptotic, and anti-angiogenic stimuli and has been used to successfully treat many different diseases [20]. We previously found that MYCN inhibition in *MYCN*-amplified NB cells resulted in upregulation of GR, and subsequent activation by dexamethasone (DEX) led to neuronal differentiation. Further, a significant decrease in tumor volume and weight was observed in all DEX- compared to vehicle-treated animals. With these data together with analysis of *TH-MYCN* mice, we demonstrated that MYCN-mediated downregulation of GR plays an important role in NB by impairing differentiation [18].

We formerly demonstrated that ERα is another downregulated NHR in *MYCN*-amplified NB patients and a prognostic factor for favorable outcome [16, 17]. However, in many tumor types, including breast cancer, overexpression and activation of ERα signalling is associated with proliferation and poor survival [21]. In contrast, during brain development, estrogen is important for neurite formation and synaptogenesis, as well as neuronal differentiation [22–24]. We found that restoration of ERα by inhibiting *miR-18a,* one member of the *miR-17 ~ 92* microRNA cluster, or ectopic ERα expression led to upregulation of NGF receptors followed by neuronal differentiation. This revealed that *MYCN-*amplified NB cells maintain an undifferentiated phenotype due to MYCN-mediated upregulation of *miR-18a*, which resulted in decrease of ERα and impairment of estrogen-induced neuronal differentiation [16, 17].

In summary, GR or ERα expression correlates with favorable prognosis in NB patients, while MYCN is related to poor outcome as it maintains an undifferentiated state by decreasing the expression of these receptors [16–18]. Activation of either GR or ERα resulted in a partial differentiation, suggesting that they could induce a more robust differentiation phenotype when triggered together.

## Material and methods

### Cell culture, cell lines, and plasmids

Cells were cultured in 1:1 of F12 and minimal essential medium (MEM) supplemented with 10% fetal bovine serum (FBS) (Life Technologies, Waltham, MA, USA), 0.5% GlutaMax, 100 units/ml penicillin, 100 μg/ml streptomycin (Sigma, St. Louis, MO, USA), and non-essential amino acids (Gibco, Life Technologies, Waltham, MA, USA) in a humidified environment at + 37 °C and 5% CO_2_.

SK-N-BE(2)-GR (from here on referred to as BE(2)-GR) cells with ectopic expression of the human *GR (NR3C1)* gene were previously generated [18]. To obtain the *GR (NR3C1)* and *ERα (ESR1)* double expressing cells, BE(2)-GR cells were transduced with a lentivirus carrying the *CD511B-1* vector (System Biosciences, Palo Alto, CA, USA) containing the human *ERα* cDNA under the control of the CMV7 promoter or a lentivirus with the corresponding empty vector (EV) as control, generating BE(2)-GR + ERα and BE(2)-GR + EV cells, respectively. Similarly, SK-N-AS and SH-SY5Y GR-overexpressing cells carried pLV-Puro-CMV-*hNR3C1* and pLV-Hygro-CMV-*EV* while GR + EV and GR + ERα IMR32 and SMS-KCN69n (from here referred to KCN69n) were generated by transduction with lentiviruses carrying pLV-Puro-CMV-*hNR3C1* and pLV-Hygro-CMV-*hESR1* or the corresponding empty vector (EV) (VectorBuilder Inc.). We used 4 μg/ml of polybrene and MOI=10 for BE(2) cells, MOI = 5 for KCN69n, MOI = 3 for IMR32 and SK-N-AS, and MOI = 1 for SH-SY5Y. Stable cell lines were generated by antibiotic selection with the following concentrations: KCN69n, 3 μg/ml puromycin, 500 μg/ml hygromycin; IMR32, 0.4 μg/ml puromycin, 400 μg/ml hygromycin; SK-N-AS, 2 μg/ml puromycin and 500 μg/ml hygromycin; SH-SY5Y, 0.8 μg/ml puromycin, 400 μg/ml hygromycin. These concentrations were applied for 48 h and thereafter cells were maintained in half of the initial concentrations of puromycin and/or hygromycin.

### Treatment of cells

All treatments were performed in media with charcoal stripped FBS (Life Technologies, Waltham, MA, USA) to avoid activation of any of the NHRs due to the presence of their ligands in regular FBS. Throughout the study, 10 nM of E2 (17-β-estradiol), 100 nM DEX (dexamethasone), and 0.5 μM of ATRA (all-*trans* retinoic acid) were used if not indicated differently for BE(2) cells, 20 nM of E2, 200 nM DEX and 1 μM of ATRA for KCN69n and IMR-32 cells, and 100 nM DEX and 5 μM of ATRA for SK-N-AS and SH-SY5Y cells. In all experiments, ethanol was used as control for E2, DEX, and the combination of E2 + DEX, dimethyl sulfoxide (DMSO) for ATRA treatment, and ethanol+DMSO as control for the E2 + DEX + ATRA triple combination. All ligands were purchased from Sigma, St. Louis, MO, USA.

### Colony formation assay

Cells were cultured in six-well plates at a density of 100 cells per well. Ligands and vehicles were added after 3 days in culture as indicated, replenished every 3 days, and incubated for 10 days. Colonies were stained using 0.5% crystal violet solution (SERVA Electrophoresis GmbH, Heidelberg, Germany). Images were captured and colonies were quantified using ImageJ software [25].

### WST-1 viability assay for determination of IC_50_ values

For calculating the IC_50_ of the individual ligands 1 × 10^4^ cells were seeded in 96 well-plates and treated for 72 h with a range of concentrations of the different compounds: E2 (1 nM-100 nM), DEX (20 nM-1000 nM), and ATRA (0.5 μM-100 μM). For assessing the effect of combining these ligands, the following concentrations were applied: E2 (10 nM and 20 nM) + DEX (100 nM and 250 nM) and E2 (10 nM and 20 nM) + DEX (100 nM and 250 nM) + ATRA (0.5 μM and 2 μM). For measurement of cell viability, 10 μl of 2-(4-Iodophenyl)-3-(4-nitrophenyl)-5-(2,4-disulfophenyl)-2H-tetrazolium (WST-1) solution (Roche, Basel, Switzerland) was added per well. After 1 h incubation, absorbance was measured at 480 nm using a LUMIstar Omega plate reader (BMG Labtech, Ortenberg, Germany).

### Western blot analysis

Whole-cell lysates were prepared using RIPA lysis buffer containing a protease and phosphatase inhibitor cocktail (both from Thermo Fisher Scientific, MA, USA). Samples were boiled in 1× Laemmli buffer at + 95 °C for 5 min and separated by sodium dodecyl sulfate-polyacrylamide gel electrophoresis (SDS-PAGE) (Bolt™ 4-12% Bis-tris Plus, Invitrogen, Waltham, MA, USA). Following electrophoresis, the separated proteins were transferred to nitrocellulose membranes (Trans-Blot Turbo Transfer Pack, Cat No. 1704159, Bio-Rad, CA, USA). Membranes were stained with Ponceau to verify equal transfer. After blocking in 5% non-fat milk, membranes were probed at + 4 °C overnight with the following primary antibodies: rabbit anti-GR (1:1000, D8H2), rabbit anti-ERα (1:1000, D8H8), rabbit anti-p75^NTR^ (1:1000, D4B3), mouse anti-MYCN (1:500, sc-53,993), mouse anti-RARα (1:500, sc-515,796), rabbit anti-SCG2 (1:1000, HPA011893), rabbit anti-p75^NTR^ (1:1000, D4B3), mouse anti-βIII-tubulin (1:1000, ab7751), mouse anti-TH (1:300, 22,941), mouse anti-GFAP (1:2000, ab4648), mouse anti-c-MYC (1:500, sc-40), rabbit anti-c-MYC (1:2000, #9402), β-actin (1:10,000, SC-47775), and α-tubulin (1:10,000, SC-32293). Next day, membranes were incubated with horseradish peroxidase tagged goat anti-mouse or goat anti-rabbit secondary antibodies (P044801-2 and P044701-2 Agilent Technologies, North Billerica, MA, USA). Signals were developed using SuperSignal™ West Dura (Cat No. 34076, Thermo Fisher Scientific, MA, USA). All Western blots were carried out at least three times and were quantified using ImageJ software. The antibodies for MYCN, c-MYC (sc-40), β-actin, α-tubulin, and RARα were purchased from Santa Cruz Biotechnology (Dallas, TX, USA), anti-GR, anti-ERα, anti-p75^NTR^ and anti-c-MYC (#9402) from Cell Signaling Technology (Danvers, MA, USA), anti-βIII-tubulin and anti-GFAP from Abcam (Cambridge, UK), anti-TH from Immunostar (Hudson, WI, USA), and anti-SCG2 from Atlas Antibodies (Bromma, Sweden).

### Bright field microscopy and immunofluorescence of neurite outgrowth

BE(2)-GR + EV and BE(2)-GR + ERα cells were seeded overnight at a density of 1.2 × 10^5^ for control and ATRA, and 2.0 × 10^5^ for the E2 and DEX treatments in six-well plates containing glass coverslips. Next day, cells were exposed to the indicated ligands and incubated at + 37 °C for 7 days. Cells were fixed in 4% paraformaldehyde solution. For immunofluorescence, coverslips were incubated for 1 h at room temperature with blocking solution (5% goat serum, 0.25% Triton X-100, 1% BSA, and 0.05% sodium azide (Sigma, St. Louis, MO, USA), in PBS). For staining, coverslips were incubated overnight with mouse anti-βIII-tubulin (1:500, ab7751), rabbit anti-SCG2 (1:500, HPA011893), or mouse anti-vimentin (1:200, M0725) diluted in blocking solution at + 4 °C. Next day, samples were washed three times with PBS and incubated with AlexaFluor 594-conjugated goat anti-mouse, AlexaFluor 488-conjugated goat anti-rabbit (Invitrogen, Waltham, MA, USA) secondary antibodies, or with phalloidin Dylight TM 554 (1:500, 13054S) for 1 h at room temperature. DAPI (1:10,000) was added to stain the nuclei. After 10 min of incubation, cells were washed three times in PBS. Images were captured with a Zeiss Axiovert 200 M microscope with the Zen 2 blue edition software. Phalloidin Dylight TM 554 was purchased from Cell Signaling (Danvers, MA, USA). Neurites from three separate images from three independent experiments were quantified using ImageJ. A similar procedure was applied for KCN69n, IMR32, SK-N-AS, and SH-SY5Y cells.

### Real-time qPCR

Total RNA was isolated using TRIzol reagent (Invitrogen, Waltham, MA, USA) and extracted with DirectZol RNA miniprep kit (Qiagen, Hilden, Germany). cDNA was synthesized using iScript cDNA synthesis kit (Bio-Rad, Hercules, CA, USA) according to manufacturer’s instructions. *TRKA*, *NEFL*, *SOX2*, *NES*, *MYCN,* and *DLG2* mRNA expression was evaluated by Real-time qPCR using iTaq universal SYBR Green supermix (BioRad, Hercules, CA, USA) and performed on a StepOnePlus Real-Time PCR system (Applied Biosystems, Waltham, MA, USA) according to the manufacturer’s instructions. Samples were run in triplicate and normalized to the mRNA levels of the internal controls, *β-2-microglobulin* (*B2M*) or *β-actin*. Relative mRNA expression was calculated using the ΔΔCT method. Primer sequences used are listed below. At least three independent experiments were performed.

*TRKA*: Forward: 5’*CACTAACAGCACATCTGGAGACC*3’.

Reverse:5’*TGAGCACAAGGAGCAGCGTAG*3’.

*NEFL*: Forward: 5’*CTGGAAATCGAAGCATGCCG*3’.

Reverse: 5’*GCGGGTGGACATCAGATAGG*3’.

*SOX2*: Forward: 5’*ATGCACCGCTACGACGTGA*3’.

Reverse: 5’*CTTTTGCACCCCTCCCATTT*3’.

*NES*: Forward: 5’*CTGCTACCCTTGAGACACCTG*3’.

Reverse: 5’*GGGCTCTGATCTCTGCATCTAC*3’.

*MYCN*: Forward: 5’*GCAGAGAGGTCCTGTTTCCC*3’.

Reverse: 5’*GCCAGAGAGTCCCTTTCACC*3’.

*DLG2*: Forward: 5’*CCCAGGTCTCTGGAACCTCT*3’.

Reverse: 5’*TGCTCGATCATAGGTTTTCTTG*3’.

*Β2M:* Forward: 5’TGCTGTCTCCATGTTTGATGTATC3’.

Reverse: 5’TCTCTGCTCCCCACCTCTAAGT3’.

*β-actin*: Forward: 5’AACTCCATCATGAAGTGTGACG3’.

Reverse: 5’GATCCACATCTGCTGGAAGG3’.

### Nile red staining for lipid droplets

BE(2)-GR + EV and BE(2)-GR + ERα cells were cultured overnight at a density of 1.0 × 10^5^ cells in six-well plates containing glass coverslips. Next day, cells were exposed to the indicated ligands and incubated at + 37 °C for 72 h. Cells were fixed in 4% buffered paraformaldehyde solution. For lipid droplet staining, coverslips were incubated with Nile Red (Sigma, St. Louis, MO, USA) (1:1000) for 15 min at RT. After washing with PBS, DAPI was used (1:10,000) to stain nuclei followed by washes with PBS. Images were captured with a Zeiss Axiovert 200 M microscope using the Zen 2 blue edition software.

### Extracellular flux analysis

XFe 96 Analyzer (Seahorse Bioscience, Agilent Technologies, North Billerica, MA, USA) was employed to determine the glycolytic and mitochondrial functions in BE(2)-GR + EV and BE(2)-GR + ERα cells. To this end, extracellular acidification rates (ECAR) and oxygen consumption rates (OCR) were measured by performing Glycolysis and Cell Mito stress tests, respectively. Cells were plated at 8 × 10^3^ cells/well in 96-well plates (Seahorse cell culture micro plates) in standard cell culture medium. Next day, cells were treated with 10 nM E2, 100 nM DEX, E2 + DEX, or 0.5 μM ATRA, as well as the combination of the three ligands in charcoal stripped FBS media for 72 h. One hour prior the assay, culture medium was replaced with XF assay medium (pH 7.4; Agilent Technologies) supplemented with 2 mM glutamine (Glycolysis stress test) or 2 mM glutamine, 1 mM pyruvate and 10 mM glucose (Cell Mito stress test) and cells were incubated at + 37 °C in a CO_2_-free incubator. Oligomycin and carbonyl cyanide 4-(trifluoromethoxy) phenylhydrazone (FCCP) were applied at final doses of 1 μM. Glucose, 2-deoxyglucose (2-DG), rotenone, and antimycin A were used at the final doses recommended by the manufacturer’s instructions. Extracellular acidification rate (ECAR) and oxygen consumption rate (OCR) values were expressed as mpH/min and pmol/min, respectively. Baseline levels were normalized to protein contents. For obtaining the energy profile and metabolic potential, the baseline levels of BE(2)-GR + EV and BE(2)-GR + ERα cells were compared before and after induction of metabolic stress using 1 μM oligomycin (stressed ECAR) and 1 μM FCCP (stressed OCR).

### In vivo animal experiments

Five-week-old female NMRI-*Foxn1*^*nu*^ mice purchased from Taconic Biosciences were housed in specific pathogen free conditions where light, temperature (+ 21 °C), and relative humidity (50-60%) were controlled. Food and water were available ad libitum. For xenograft experiments, 8 × 10^6^ BE(2)-EV or BE(2)-GR cells, and 1.0 × 10^7^ BE(2)-GR + EV or BE(2)-GR + ERα cells were inoculated subcutaneously into the right flank of the mice. Tumor growth was followed daily, and tumor volume was calculated as width × length × height × 0.52. Animals were monitored every day for signs of weight loss and euthanized when tumors in the control group reached the maximum permitted volume of 1 cm^3^. At sacrifice, tumors were dissected and fixed in formaldehyde for further analysis. The procedures for all animal experiments were in accordance with the ethical principles and guidelines of Karolinska Institutet and the Swedish law. Ethical permit numbers N71/15 and 10579-2020 approved by the Ethical Committee at the Northern court of Stockholm.

### Immunohistochemistry analysis of tumor sections

For immunohistochemistry analysis, xenograft tumors were fixed in 4% paraformaldehyde and embedded in paraffin. Tumors were sectioned (5 μm thick) and stained with the EnVision Gl2 Doublestain System (Agilent Technologies, North Billerica, MA, USA) according to the manufacturer’s instructions. Tumor sections were deparaffinized and rehydrated using a gradient of xylol-alcohol solutions. Endogenous peroxidase activity was blocked by 30 min incubation in methanol-hydrogen peroxide solution and sections were boiled for antigen retrieval in sodium citrate buffer (pH 6.0), except for the staining with endomucin, for which Tris-EDTA buffer was used (pH 9.0). After blocking, samples were incubated overnight at + 4 °C with primary antibodies; rabbit anti-Ki67 (1:250, ab16667), rabbit anti-GR (1:400, D6H2L), rabbit anti-ERα (1:100, ab32063), rabbit anti-SCG2 (1:250, HPA011893), mouse anti-βIII tubulin (1:250, ab7751), mouse anti-p75^NTR^ (1:500, D4B3), or mouse anti-endomucin (1:400, eBioV.7C7) diluted in blocking solution. Next day, samples were incubated with HRP-polymer for 20 min, followed by 30 seconds-2 min incubation with 3,3′-Diaminobenzidine (DAB) solution. For nuclear staining, samples were incubated with hematoxylin (Agilent Technologies, North Billerica, MA, USA), for 2 min, dehydrated by a gradient of alcohol-xylol solutions and mounted with Roti-histokitt mounting medium (Carl Roth, Karlsruhe, Germany). Section images were taken at 40x with inverted microscope Olympus IX73. The antibody against GR was purchased from Cell Signaling Technology (Danvers, MA, USA), anti-ERα, anti-Ki67 and anti-βIII tubulin from Abcam (Cambridge, UK), anti-SCG2 from Sigma (St. Louis, MO, USA), anti-p75^NTR^ from Cell Signaling (Danvers, MA, USA), and anti-endomucin from eBioscience Affymetrix (Santa Clara, CA, USA).

### Receptor expression analysis in NB cell lines

RNA sequencing data from 39 commonly used neuroblastoma cell lines (GSE89413) [26] was analyzed to study the levels of *GR*, *ERα*, and *RARα*. The average of the log (fold change) of the expression levels (measured as fragments per kilobase of exon per million mapped fragments, FPKMs) of all samples was used to determine high and low levels. Diverging bar charts were performed using package ‘ggpubr’ in R (version 4.1.3).

### Gene expression analyses

RNA sequencing data and clinical information from 498 patient samples were obtained from the SEQC (GSE62564) [27] and microarray and clinical data from 649 patient samples from the Kocak (GSE45547) [28] NB cohorts downloaded from GEO (https://www.ncbi.nlm.nih.gov/geo/). Patients were individually divided based on low and high expression of the three different NHR genes *GR (NR3C1)*, *ERα (ESR1),* and *RARα (RARA)* by quartile expression score. The first quartile was considered as “low”, corresponding to the patients with the lowest *GR*, *ERα,* or *RARα* (Low^*GR*^, Low^*ERα*^*, *or Low^*RARα*^) mRNA expression levels, and the fourth quartile as “high”, corresponding to patients with the highest *GR*, *ERα*, or *RARα* levels (High^*GR*^*,* High^*ERα*^, or High^*RARα*^). Transcription data and clinical information of 251 patients from the Oberthuer dataset [29] were obtained from ArrayExpress platform (https://www.ebi.ac.uk/arrayexpress/) under the accession number E-MTAB-38. Patients were divided based on low and high expression of *GR*, *ERα,* and *RARα* by quartile expression score. Analysis was carried out as described above.

In the intersection of the first quartiles of *GR* and *ERα* patients with low mRNA expression levels, Low^*GR + ERα*^, we obtained 80 patients in the SEQC and 41 in the Kocak cohorts, respectively. In addition, for the overlap of the fourth quartiles of *GR* and *ERα* with high mRNA expression*,* High^*GR + ERα*^, 74 patients were identified in the SEQC dataset and 46 in the Kocak cohort. Two samples in the Kocak cohort lacked survival information thus we used 40 Low^*GR + ERα*^ and 45 High ^*GR *^^+ *ERα*^ patients. Using the SEQC dataset, patients with High^*GR + ERα*^ and Low^*GR + ERα*^ were also divided according to their *MYCN*-status, amplified (MNA) or non-amplified (NMNA), respectively. This resulted in 74 High^*GR + ERα*^ NMNA, 29 Low^*GR + ERα*^ NMNA, 50 Low^*GR + ERα*^ MNA while no single MNA patient with High^*GR + ERα*^. The *MYCN* status was unknown for one of the patients included in the Low^*GR* + *ERα*^ group and thus 79 patients were used in the analysis. Additionally, the levels of *GR*, *ERα,* and *RARα* were compared between the total number of MNA *versus* NMNA group of patients.

To study all three NHRs, the intersection of the first and fourth quartiles of triple mRNA expression of *GR*, *ERα,* and *RARα* resulted in 24 patients with high expression, High^*GR + ERα + RARα*^, and 36 with low expression, Low^*GR + ERα + RARα*^, in the SEQC cohort, while ten High^*GR + ERα + RARα*^ and eight Low^*GR + ERα + RARα*^ patients were identified in the Kocak dataset.

Expression levels of the genes encoding the neuronal differentiation marker tyrosine hydroxylase (TH), secretogranin 2 (SCG2), and the neurotrophic receptor (p75^NTR^), also known as nerve growth factor receptor (NGFR) were analyzed in the High^*GR + ERα*^ and Low^*GR + ERα*^, as well as in the High^*GR + ERα + RARα*^ and Low^*GR + ERα + RARα*^ mRNA expression groups both of the SEQC as well as Kocak cohorts. Analysis of the differential processes affected between the High^*GR + ERα*^ and Low^*GR + ERα*^ mRNA expression groups from the SEQC dataset was performed using GSEA software (v4.0.3). Gene sets used for enrichment were obtained from C2: curated sets collection (c2.all.v7.2.symbols.gmt) (Additional File 1), and C5: ontology gene sets collection (c5.all.v7.2.symbols.gmt) (Additional File 2).

To determine the expression profile of genes of interest in normal and embryonic adrenal tissue, the data matrix of the Suntsova dataset (GSE96631) [30] was downloaded from the GEO database (https://www.ncbi.nlm.nih.gov/geo/). Expression values of each gene were plotted into a heatmap. Among the 2227 probes in the platform GPL22167, *NR3C1*, *NGFR*, *TUBB3,* and *ERBB3* were presented as indicated.

### Single-nuclei transcriptome analyses

The lists of genes significantly up-regulated in ten cell clusters (*i.e.* Progenitor-hC1, Macrophages-hC2, Cortex-hC3, -hC5, -hC8, and -hC9, Chromaffin-hC4, Endothelial -hC6, Mesenchymal-hC7, and T-cells-hC10) from three post-natal adrenal glands were obtained from Bedoya-Reina et al. 2021 [31]. These lists were generated by computing gene-expression differences on the PAGODA-standardized expression using Benjamini-Hochberg corrected one-tailed Welch’s *t*-tests. This dataset analyzed whether the PAGODA-standardized expression of genes (including *NR3C1,* encoding for GR, *RARA* for RARα, and *ESR1* for ERα) were significantly up-regulated in cells of each post-natal adrenal gland cluster in comparison with cells of the other clusters.

Gene expression in developing human adrenal glands was studied with the single cell sequencing data reported by Dong et al. (2020) [32] on differentially expressed genes in sympathoadrenal cell types and states in human fetal adrenal gland (selecting genes with an adjusted *p* < 0.01). Pseudo-time progression on post-natal adrenal gland cells was computed as previously conducted [31]. Briefly, velocities were estimated with scVelo [33, 34] with a minimum number of shared read counts of 40 using the top 600 genes, in the gene counts obtained by Bedoya-Reina et al. (2021) [31].

### Statistical analysis

All in vitro and in vivo experiments were analyzed by Student’s *t*-test or Mann-Whitney test, as indicated in the figure legends. All data is presented as mean ± standard deviation (SD) of at least three independent experiments. Significance is highlighted with *, **, ***, and **** indicating *p* < 0.05, *p* < 0.01, *p* < 0.001, and *p* < 0.0001, respectively.

Patient data from the SEQC, Kocak, Oberthuer, and Suntsova datasets were analyzed using GraphPad Prism software (v.8). *T*-test was used for statistical analysis of gene expression with *, **, ***, and **** indicating *p* < 0.05, *p* < 0.01, *p* < 0.001, and *p* < 0.0001. Significance of overall survival (OS) curves was analyzed by Log-rank test. *T*-test was used for statistical analysis of gene expression with *, **, ***, and **** indicating *p* < 0.05, *p* < 0.01, *p* < 0.001, and *p* < 0.0001. The significance threshold for gene enrichment analysis using GSEA (v4.0.3) was set to an FDR *q*-value of 0.05. Normalized enrichment score (NES) was defined as actual ES/mean (ES against all permutations of the dataset).

The lists of genes from the single-nuclei analysis were generated by computing gene-expression differences on the PAGODA-standardized expression using Benjamini-Hochberg corrected one-tailed Welch’s *t*-tests. For the gene expression in developing human adrenal glands, we selected genes with an adjusted *p* < 0.01.

## Results

### Ectopic GR expression induced differentiation and reduced tumorigenesis

We first analyzed the effect of a synthetic ligand of GR, dexamethasone (DEX), and the RARα ligand all-*trans* retinoic acid (ATRA) on the viability of BE(2) cells stably expressing GR, named BE(2)-GR, and the empty vector carrying control BE(2)-EV, previously generated [18]. Following treatment with ATRA in BE(2)-GR cells, viability increased but no significant changes were found after treatment with either DEX or DEX + ATRA (Supplementary Fig. 1A).

**Fig. 1:**
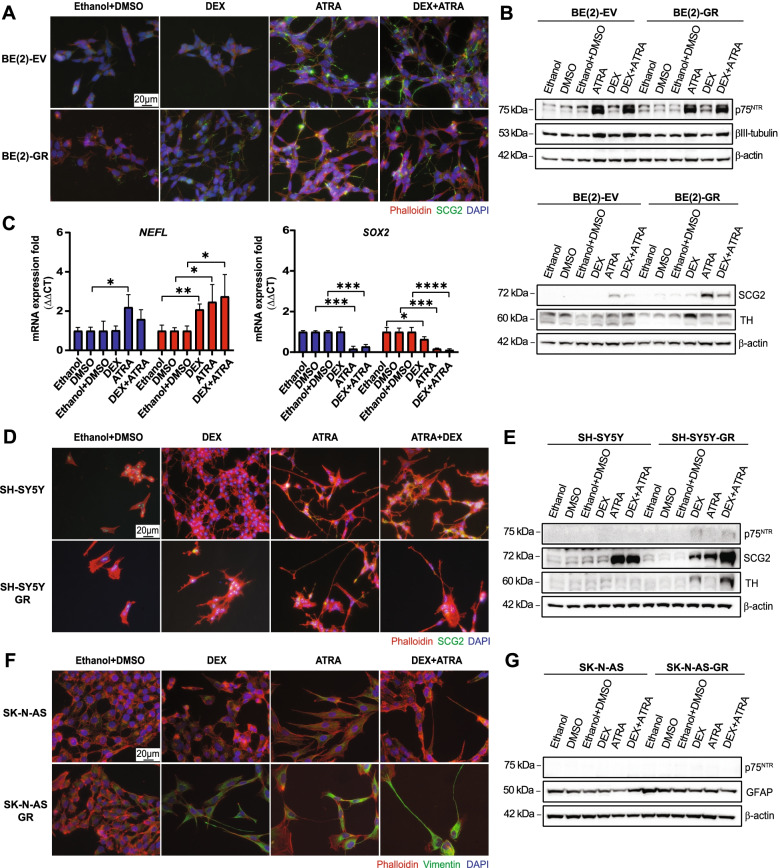
Expression of GR reduced proliferation, induced neuronal and glial differentiation*,* and decreased tumor growth. **A** Immunofluorescence staining of BE(2)-EV and BE(2)-GR cells after ligand treatment. Green = SCG2; red = Phalloidin; blue = DAPI. **B** Western blot of indicated markers in BE(2)-EV and BE(2)-GR cells following incubation with ligands. Please note the different loading order on the two membranes. **C** RT-qPCR of *NEFL* and *SOX2* in BE(2)-EV and BE(2)-GR cells following ligand treatment. *B2M* was used as housekeeping gene. Statistical analysis: *t*-test with *, **, ***, and **** indicating *p* < 0.05, *p* < 0.01, *p* < 0.001, and *p* < 0.0001, respectively. Data of the fold mRNA expression is presented as mean ± SD. **D** Immunofluorescence staining of SH-SY5Y parental and GR expressing cells after incubation with ligands. Green = SCG2; red = Phalloidin; and blue = DAPI. **E** Western blot of neural differentiation markers in SH-SY5Y parental and GR expressing cells following ligand treatment. **F** Immunofluorescence staining of SK-N-AS parental and GR expressing cells after incubation with ligands. Green = Vimentin; red = Phalloidin; blue = DAPI. **G** Western blot of neural and glial differentiation markers in SK-N-AS parental and GR expressing cells following ligand treatment. All experiments were carried out during seven days. Ethanol was used as control for DEX, DMSO for ATRA, and ethanol + DMSO for the combination. Scale bars represent 20 μm. β-actin was used as loading control and molecular weight markers are shown to the left. All results are representative of at least three independent experiments

Well-differentiated tumor cells are usually linked to a less aggressive phenotype [35]. We therefore asked whether DEX stimulated differentiation of BE(2)-GR cells. Retinoic acid (RA) is known for its differentiation mediating ability in NB [36] and was thus used as positive control. As expected, ATRA triggered neurite-like outgrowth, a morphological sign of neuronal differentiation. Activation of GR with DEX showed a similar phenotype and the DEX + ATRA combination slightly increased neurite formation compared to the single treatments in BE(2)-GR cells (Fig. 1A, Supplementary Fig. 1B and Supplementary Fig. 1C). In contrast, only ATRA provoked differentiation in BE(2)-EV cells. The levels of GR and RARα were analyzed by Western blot to determine expression changes upon treatment. We found that GR levels declined in BE(2)-GR cells incubated with DEX (Supplementary Fig. 1D), consistent with a well described negative feedback loop [18, 37]. The abundance of RARα was similar in both cell lines, and ATRA slightly increased the levels in BE(2)-GR cells while ATRA, DEX, or the combination led to a decrease in control cells (Supplementary Fig. 1D). Reduction of MYCN levels is a well-known effect during neuronal differentiation [38, 39]. Hence, MYCN protein was downregulated in BE(2)-EV cells treated with either ATRA or DEX + ATRA and mRNA levels were slightly reduced (Supplementary Fig. 1D, E). In BE(2)-GR cells, single treatment with ATRA reduced *MYCN* mRNA expression (Supplementary Fig. 1F). The neuronal differentiation marker tyrosine hydroxylase (TH) was elevated in BE(2)-GR cells upon DEX exposure, with a slight increase in the p75 neurotrophin receptor (p75^NTR^), while no changes were observed in secretogranin 2 (SCG2) or βIII-tubulin (Fig. 1B and Supplementary Fig. 1E). ATRA treatment alone or in combination with DEX resulted in an upregulation of differentiation markers, particularly in SCG2 in both cell lines. Expression analysis of mRNA levels of additional differentiation markers including *Neurofilament L* (*NEFL*) and *Tropomyosin receptor Kinase A* (*TRKA*), as well as the progenitor markers *Nestin* (*NES*) and *Sex determining region Y-box 2* (*SOX2*), were performed by RT-qPCR to validate the induction of neuronal differentiation upon GR and RARα activation (Fig. 1C and Supplementary Fig. 1F). Treatment with DEX resulted in upregulated expression of *NEFL* and *TRKA* only in BE(2)-GR cells, while ATRA increased their levels in both cell lines. Both *NES* and *SOX2* were significantly downregulated in BE(2)-GR cells in all conditions compared to control while in BE(2)-EV cells only *SOX2* was significantly reduced when ATRA was added.

Moreover, we extended the differentiation studies by overexpressing GR in two additional cell lines, SH-SY5Y and SK-N-AS, both non-*MYCN*-amplified but expressing c-MYC. First, we assessed the protein levels of GR, RARα, and c-MYC after treatment with DEX, ATRA, or their combination (Supplementary Fig. 2A). In SH-SY5Y parental cells, GR protein levels were barely detected by Western blot analysis, while they were highly expressed in SH-SY5Y-GR cells, with all treatments. Furthermore, c-MYC levels decreased upon ATRA or DEX + ATRA in both cell lines while RARα was barely detectable in any condition in SH-SY5Y-GR cells (Supplementary Fig. 2A, B). We observed low GR expression in parental SK-N-AS. Exposure to DEX or DEX+ATRA reduced GR levels in both SK-N-AS parental and GR-overexpressing cells. Moreover, all treatments reduced RARα expression in both cell lines. c-MYC was downregulated in parental cells upon DEX + ATRA, and with DEX in SK-N-AS-GR cells (Supplementary Fig. 2A, B).

In SH-SY5Y parental and SH-SY5Y-GR cells, ATRA exposure resulted in neurite outgrowth, while DEX alone induced mild differentiation observed as a moderate neurite outgrowth in GR-overexpressing cells (Fig. 1D and Supplementary Fig. 2C). Moreover, SCG2 was increased in all ATRA conditions, while the other markers analyzed were barely detectable in parental cells (Fig. 1D, E and Supplementary Fig. 2D). In the SY5Y-GR cells, SCG2 was enhanced upon treatments, while TH increased only with DEX or DEX + ATRA, and p75^NTR^ only slightly with all conditions (Fig. 1D, E and Supplementary Fig. 2D).

SK-N-AS cells are well known to differentiate into the glial lineage. Therefore, two glial differentiation markers, vimentin (VIM) and glial fibrillary acidic protein (GFAP), were analyzed. Treatment with DEX slightly increased vimentin abundance in parental SK-N-AS, and this effect was stronger in SK-N-AS-GR cells (Fig. 1F). All-*trans* retinoic acid (ATRA) and the combination with DEX induced vimentin expression in both cell lines, notably more pronounced in the GR-overexpressing cells, also evidenced by morphological changes (Fig. 1F), while GFAP protein levels were not changed (Fig. 1G and Supplementary Fig. 2E). As expected, the neural differentiation marker p75^NTR^ was not expressed either in the parental or the GR-overexpressing cells, verifying the differentiation towards the glial lineage instead of neuronal of SK-N-AS cells.

**Fig. 2:**
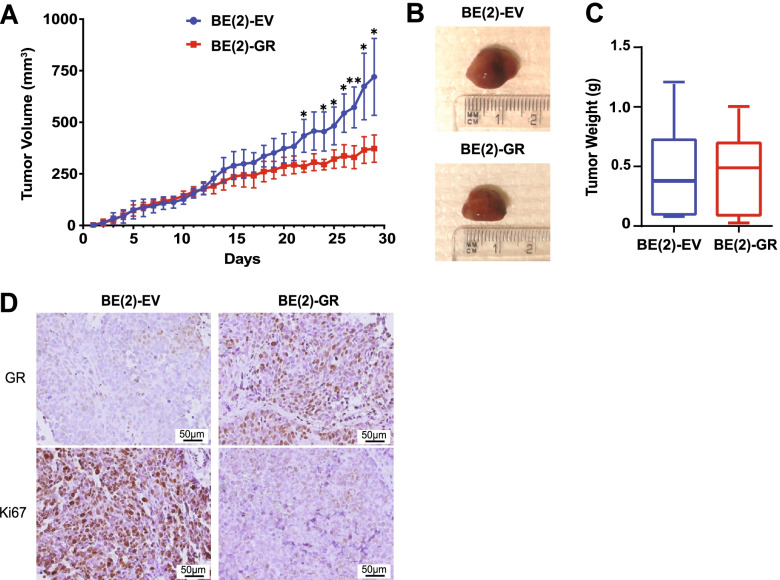
Expression of GR decreased tumor growth. **A** Tumor volume in mice inoculated with BE(2)-EV (*n* = 6) and BE(2)-GR (*n* = 7) cells. Growth was followed until the control group reached the ethical endpoint volume of 1 cm^3^. Statistical analysis: *t*-test with * and ** indicating *p* < 0.05 and *p* < 0.01, respectively. Data is presented as mean ± SD. **B** Representative photos of xenograft tumors at experimental endpoint with ruler. **C** Tumor weight at experimental endpoint. Statistical analysis: *t*-test. Data is presented as mean ± SD. **D** Images of immunohistochemistry analysis of BE(2)-EV and BE(2)-GR derived tumors stained with anti-GR and anti-Ki67 antibodies. Representative pictures from at least five independent stainings per condition. Scale bars indicate 50 μm

To evaluate possible differences in tumor growth in vivo, we performed a xenograft experiment comparing BE(2)-EV and BE(2)-GR cells. Tumor volume was significantly decreased in mice injected with cells overexpressing GR compared to those generated by control cells (Fig. 2A, B*,* and Supplementary Fig. 3A). However, there was no significant difference in weight at experimental endpoint, since tumors from BE(2)-EV were filled with fluid with signs of necrosis, while BE(2)-GR derived tumors exhibited a solid texture (Fig. 2C). Analysis of tumor sections confirmed the presence of GR protein and low expression of Ki67, a proliferation marker associated with aggressiveness, in BE(2)-GR-derived tumors in contrast to in control tumors (Fig. 2D).

Together, our results showed that activation of GR-expressing cells resulted in increased expression of neuronal differentiation markers and neurite outgrowth, and in reduced tumor burden in vivo*.*

### Triple activation of ERα, GR, and RARα induced differentiation

Combination treatments, *i.e.,* therapeutic interventions with a cocktail of two or more drugs, is a cornerstone of cancer therapy. The amalgamation of anti-cancer medicines improves effectiveness compared to monotherapy since it targets key pathways in a characteristically additive or synergistic manner [40].

We found that treatment with 17-β-estradiol (E2) impaired the tumorigenic potential of NB cells overexpressing ERα [17] and that GR overexpression led to reduced tumorigenesis both in vitro and in vivo (Figs. 1 and 2, Supplementary Figs. 1-3) [18]. Although both effects were robust, they did not result in complete differentiation. To assess whether concomitant overexpression of GR and ERα would promote a stronger differentiation phenotype, we generated BE(2)-GR + EV and BE(2)-GR + ERα cells using lentiviral transduction (Supplementary Fig. 4A) and performed in vitro and in vivo assays. All-*trans* retinoic acid (ATRA) was used as positive control for neuronal differentiation and the impact of RARα activation on differentiation triggered by GR and ERα was analyzed.

GR and ERα expression were validated in BE(2)-GR + EV and BE(2)-GR + ERα cells by Western blot analysis (Supplementary Fig. 4A) and the IC_50_ value for each ligand was calculated. While DEX showed similar IC_50_ values in both cell lines, E2, as expected, barely affected viability of BE(2)-GR + EV cells (Supplementary Fig. 4B). The IC_50_ values for ATRA were similar in GR + ERα overexpressing and control cells. To evaluate possible cumulative effects upon activation of two or three ligands, we performed assays of single and combination treatments. All concentrations employed were below the IC_50_ values and ATRA was used in a significantly lower concentration (0.5 μM) than in many other studies (10 μM) [41, 42]. Single treatments using any of the concentrations analyzed did not affect cell viability (Supplementary Fig. 4C). Interestingly, 0.5 μM ATRA significantly increased viability of BE(2)-GR + ERα cells, while only slightly in BE(2)-GR + EV, similar as observed in BE(2)-GR cells (Supplementary Fig. 1A). While the triple combination of 10 nM E2 + 100 nM DEX + 0.5 μM ATRA did not affect cell viability in BE(2)-GR + EV cells, a reduction to 52% occurred when increasing the concentrations to 20 nM E2 + 250 nM DEX + 2 μM ATRA (Supplementary Fig. 4C). In BE(2)-GR + ERα cells, the incubation with E2 + DEX resulted in mild reduction of cell viability, which decreased to 63% upon triple combination using low concentrations of ligands, and it was strikingly reduced to 26% with higher concentrations (20 nM E2 + 250 nM DEX+ 2 μM ATRA) (Supplementary Fig. 4C). This demonstrated that ATRA decreased viability when combined with E2 + DEX in an additive and dose-dependent manner, even though ATRA alone at low concentration increased the percentage of viable cells.

Next, we examined the colony formation potential of BE(2)-GR + EV and BE(2)-GR + ERα cells and found that the latter formed significantly fewer colonies compared to control cells (Supplementary Fig. 4D). Treatment with ATRA or the triple combination led to formation of small, highly differentiated colonies that easily detached from the plate. Neither E2, DEX, nor E2 + DEX addition led to significant differences in colony number in control cells, while in BE(2)-GR + ERα cells numbers were reduced with either E2 or E2 + DEX.

Given the decrease in colonies between BE(2)-GR + ERα and control cells, we enquired whether activation of the three receptors together would trigger a stronger differentiation. To this end, we analyzed neurite outgrowth after single, double, or triple treatments. As expected, neurite-like protrusions appeared in BE(2)-GR + EV cells upon DEX and ATRA addition, whereas the morphology did not change with E2 alone (Supplementary Fig. 5A). The combined E2 + DEX cocktail also resulted in neurite outgrowth while the triple combination further enhanced neuronal differentiation. In contrast, all treatments promoted neurite outgrowth in BE(2)-GR + ERα cells, an effect that was most robust upon the triple condition (Supplementary Fig. 5A, B).

**Fig. 3:**
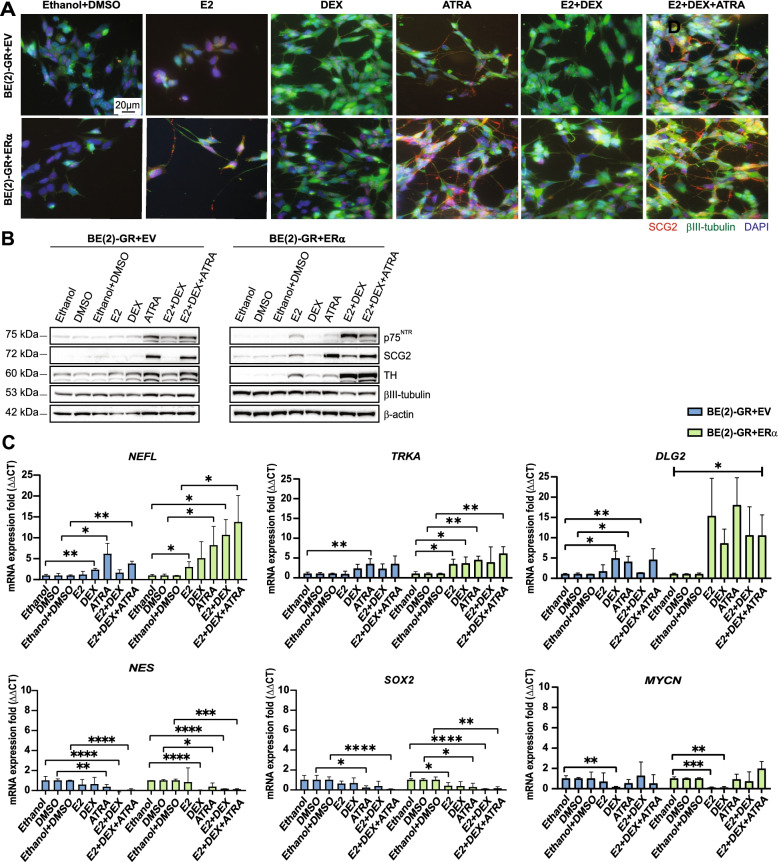
Triple activation of GR, ERα, and RARα enhanced neuronal differentiation in BE(2) cells. **A** Immunofluorescence staining after ligand treatment. Green = βIII-tubulin; red = SCG2; blue = DAPI. Scale bars represent 20 μm. **B** Western blot analysis of neural differentiation markers in BE(2)-GR + EV and BE(2)-GR + ERα cells following incubation with ligands. β-actin was used as loading control. Molecular weight markers shown to the left. **C** RT-qPCR of *NEFL, TRKA, DLG2, NES*, *SOX2,* and *MYCN* in BE(2)-GR+EV and BE(2)-GR-ERα cells following ligand treatment. *B2M* was used as housekeeping gene. Statistical analysis: *t*-test with *, **, ***, and **** indicating *p* < 0.05, *p* < 0.01, *p* < 0.001, and *p* < 0.0001, respectively. The brackets in the *DLG2* graph represent significance of all the experimental conditions *versus* their controls. Data of the mRNA expression fold is presented as mean ± SD of a minimum of three independent replicates. In all experiments, 10 nM E2, 100 nM DEX, or 0.5 µM ATRA were used with ethanol as control for E2, DEX, and E2 + DEX, DMSO for ATRA, and ethanol + DMSO for the triple combination. All experiments were carried out during seven days and performed at least three times

Neuronal differentiation was validated by immunofluorescence staining of the differentiation markers βIII-tubulin and SCG2. In control cells, βIII-tubulin increased upon exposure to DEX, ATRA, E2 + DEX, or E2 + DEX + ATRA, while SCG2 protein abundance was mainly induced with ATRA or the triple cocktail (Fig. 3A). Notably, in BE(2)-GR + ERα cells, single activation by any of the ligands resulted in elevation of both markers compared to control treatments. As observed, incubation with E2, ATRA, E2 + DEX, or E2 + DEX + ATRA, led to pronounced elongated neurite protrusions. Morphological changes also occurred in BE(2)-GR-EV cells albeit at a more modest level (Fig. 3A).

We confirmed that GR levels decreased following DEX treatment in both cell types (Supplementary Fig. 5C). In addition, BE(2)-GR + ERα cells showed higher RARα levels, which remained unchanged upon any treatment compared to control cells, where expression was decreased after incubation with E2, DEX, E2 + DEX, or the triple combination. None of the treatments affected MYCN protein expression in BE(2)-GR + EV cells, but RT-qPCR analysis revealed a significant downregulation of the *MYCN* gene upon DEX exposure (Supplementary Fig. 5C, D and Fig. 3C). Similarly, in BE(2)-GR + ERα cells, a considerable reduction in *MYCN* mRNA was observed after E2 or DEX exposure, although we did not detect any changes in protein levels. Note that the previous results included analysis on protein and mRNA abundance. A caveat of this approach is that mRNA and protein levels might not necessarily be correlated [43].

Moreover, we analyzed protein expression of the neuronal differentiation markers TH, SCG2, p75^NTR^, and βIII-tubulin by Western blot. Treatment with E2 + DEX slightly increased TH levels in control cells. In addition, ATRA alone or in combination with E2 + DEX upregulated all four markers. In BE(2)-GR + ERα cells, activation of ERα increased p75^NTR^, SCG2, and TH, and levels were robustly potentiated with triple treatment (Fig. 3B and Supplementary Fig. 5D).

Additionally, we confirmed neuronal differentiation by analyzing mRNA expression of other known neuronal differentiation and progenitor markers. Increased *NEFL* and *TRKA* were observed in all conditions with DEX or ATRA in BE(2)-GR + EV cells, whereas a downregulation in *SOX2* and *NES* occurred upon ATRA and the double (E2 + DEX) and triple combinations. Likewise, all treatments increased the abundance of neuronal differentiation markers and diminished the levels of the progenitor markers in BE(2)-GR + ERα cells (Fig. 3C). The *Discs Large MAGUK Scaffold Protein 2 (DLG2)* gene was recently reported to regulate the differentiation phenotype induced by ATRA, and proposed as a tumor suppressor candidate in NB [44]. Our RT-qPCR analysis showed that all treatments, except E2 in BE(2)-GR + EV cells, elevated *DLG2* levels in accordance with our differentiation results (Fig. 3C).

**Fig. 4:**
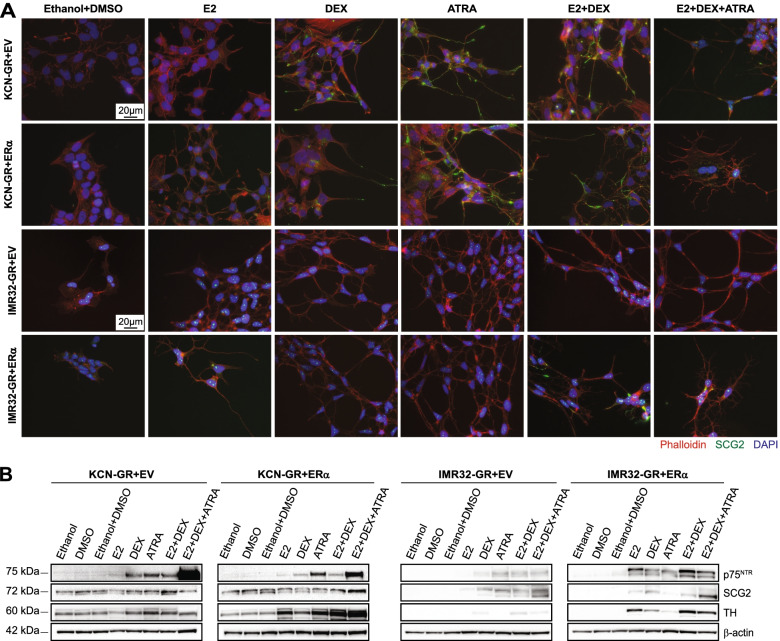
Triple activation of GR, ERα, and RARα enhanced neuronal differentiation in KCN69n and IMR32 cells. **A** Immunofluorescence staining after ligand treatment. The upper two panels show KCN69n and the lower two panels IMR32 cells. Green = SCG2; red = Phalloidin; blue = DAPI. Scale bars represent 20 μm. **B** Western blot analysis of neural differentiation markers in KCN-GR + EV, KCN-GR + ERα, IMR32-GR + EV, and IMR32-GR + ERα cells following incubation with ligands. Molecular weight markers shown to the left and β-actin was used as a loading control. Cells were treated with 20 nM E2, 200 nM DEX, 1 μM ATRA, or the combination of either 20 nM E2 + 200 nM DEX, or 20 nM E2 + 200 nM DEX + 1 μM ATRA for seven days. Experiments were performed at least three times

Furthermore, to validate the phenotype achieved by activation of the three NHRs, we expanded our analysis to two additional *MYCN*-amplified NB cell lines, IMR32 and KCN69n, both overexpressing GR + EV and GR + ERα. Equally to in BE(2) cells, neurite outgrowth was observed upon DEX, ATRA, double, and triple combination in GR + EV cells, as well as with all treatments in GR + ERα cells, in particular with E2 + DEX + ATRA in both IMR32-GR-ERα and KCN-GR + ERα (Supplementary Fig. 6A). These results were supported by phalloidin and SCG2 staining (Fig. 4A). Moreover, MYCN levels were significantly reduced with the double and triple combination in KCN-GR + ERα cells (Supplementary Fig. 6B, C). Notably, GR protein levels were downregulated upon all conditions containing DEX in GR + EV cells, and RARα expression diminished in all treatments. In contrast, all ligands increased ERα levels in KCN-GR + ERα cells (Supplementary Fig. 6B). Evaluation of the neuronal differentiation markers showed that TH was enhanced with DEX or ATRA in KCN-GR + EV and in all conditions in the double overexpressing cells. The levels of p75^NTR^ were robustly increased after the triple combination in both cell lines while the change in SCG2 was only minor (Fig. 4B and Supplementary Fig. 6C).

IMR-32-GR+ERα cells showed the most robust differentiation phenotype of all cells analyzed, with a stronger increase in all neural differentiation markers after E2 + DEX or E2 + DEX + ATRA treatment (Fig. 4A, Supplementary Fig. 6A and 7B). In IMR32-GR + EV, SCG2 levels were elevated when treated with E2 + DEX + ATRA while TH was increased with both the double and triple combination. Furthermore, while no changes were observed in MYCN protein (Supplementary Fig. 7A, B), GR levels increased in both cell lines and ERα abundance was elevated in GR + ERα cells upon treatment. The endogenous protein levels of the receptors used in this study, GR, ERα, and RARα are shown in Supplementary Fig. 7C. Moreover, using RNAseq data from 39 common NB cell lines, we analyzed the expression of the three receptors and presented data as higher or lower than the average level in all samples. All cells used in our study were present except KCN69n. The levels of *GR* (*NR3C1*) were higher than the average in SH-SY5Y cells followed by SK-N-AS, while they were under the average in SK-N-BE(2) and IMR32, with the lowest levels in the latter. In comparison, we only detected GR protein in SK-N-AS cells by Western blot (Supplementary Fig. 2A and Supplementary Fig. 7D). All cells showed lower expression of *ERα* (*ESR1*) than average (Supplementary Fig. 7D). In accordance, we did not detect *ERα* levels in any of the cells (Supplementary Fig. 4A, 5C, 6B, 7A and 7C). For *RARα* (*RARA*), mRNA levels were high in SK-N-AS, similar to average in SH-SY5Y, and lower than average in SK-N-BE(2) and IMR32 (Supplementary Fig. 7D). However, we detected higher levels of RARα protein in the *MYCN*-amplified cells BE(2), IMR32, and KCN69n, compared to the non-*MYCN*-amplified cells (Supplementary Fig. 7C).

In summary, simultaneous activation of GR and ERα lead to reduced viability and colony forming ability, accompanied by a stronger induction of neurite outgrowth and neuronal differentiation markers. Expression data revealed that cells with *MYCN*-amplification have lower levels of NHRs receptors than non-*MYCN* amplified cells, supporting the notion that MYCN inhibits their expression.

### NHR activation increased glycolytic capacity and induced lipid droplet accumulation

**Fig. 5:**
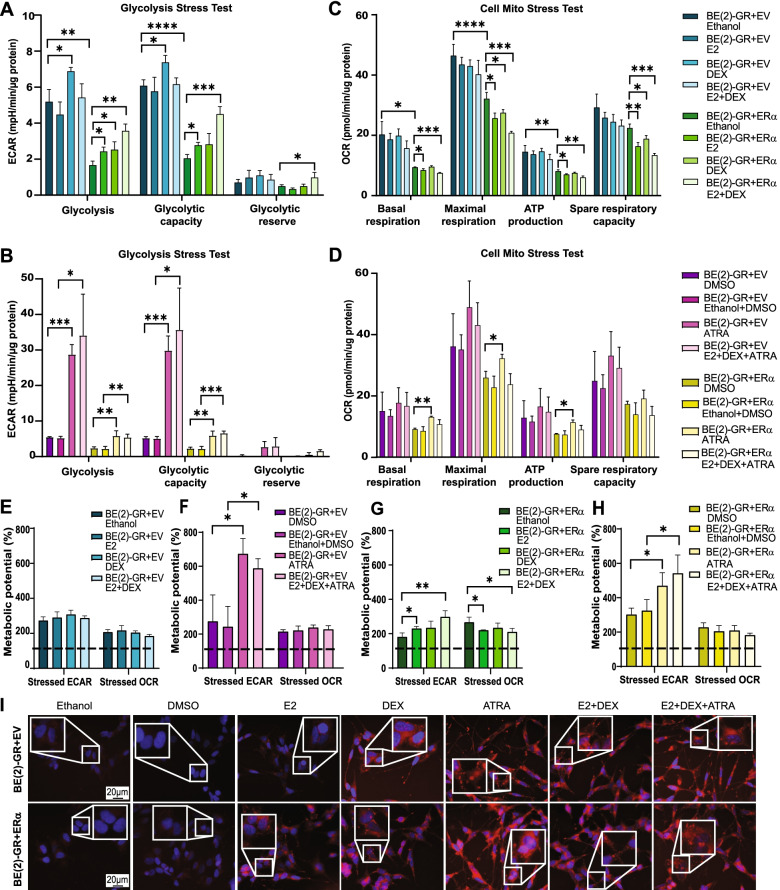
Activation of GR, ERα, and RARα resulted in metabolic reprogramming. **A-B** Quantification of extracellular acidification rate (ECAR) in BE(2)-GR + EV and BE(2)-GR + ERα cells treated with **A)** ethanol, E2, DEX or E2 + DEX, or **B)** DMSO, ethanol + DMSO, ATRA or E2 + DEX + ATRA. **C-D** Quantification of oxygen consumption rate (OCR) in BE(2)-GR + EV and BE(2)-GR + ERα cells treated with **C)** DMSO, ethanol + DMSO, ATRA or E2 + DEX + ATRA, or **D)** DMSO, ethanol + DMSO, ATRA or E2 + DEX + ATRA. **E-F** Increase in metabolic potential compared to baseline (100%, black dotted lines) in control cells exposed to oligomycin (stressed ECAR) or FCCP (stressed OCR) after incubation with **E)** ethanol, E2, DEX, E2 + DEX, or **F)** DMSO, ethanol + DMSO, ATRA, or E2 +  DEX + ATRA. **G-H** Increase in metabolic potential compared to baseline (100%, black dotted lines) in BE(2)-GR + ERα cells exposed to oligomycin (stressed ECAR) or FCCP (stressed OCR) after treatment with **G)** DMSO, ethanol + DMSO, ATRA, E2 + DEX + ATRA, or **H)** DMSO, ethanol + DMSO, ATRA, or E2 + DEX +  ATRA. **I)** Lipid droplets stained by Nile Red in control or BE(2)-GR + ERα cells treated with E2, DEX, ATRA, the combination of E2 + DEX, or E2 + DEX + ATRA. Representative images from three independent experiments. Scale bars indicate 20 μm. All experiments in **A**-**I** were performed at 72 h with 0.5 μM ATRA, 10 nM E2, and 100 nM DEX. Data is presented as mean ± SD of three independent experiments; statistical analysis: *t*-test with *, **, ***, and **** indicating *p* < 0.05, *p* < 0.01*, p* < 0.001, and *p* < 0.0001

Cancer cells reprogram their metabolism to maintain proliferation [45, 46]. We have previously shown that the glycolytic and oxidative functions were impaired in *MYCN*-amplified NB cells overexpressing ERα [17]. To examine a putative cumulative effect of GR and ERα activation on the metabolic phenotype, we employed the Agilent Extracellular Flux Analyzer (Supplementary Fig. 8A, B) [47, 48]. We found that cells expressing GR were more glycolytic and, in addition, had a higher oxidative phosphorylation (OXPHOS) capacity than those co-expressing GR and ERα (Fig. 5A, B). Incubation with DEX increased the glycolytic parameters in control cells. As expected, both E2 and DEX single treatments raised glycolysis in BE(2)-GR + ERα cells, and it was further increased upon their combination (Fig. 5A). Similarly, treating with ATRA or the triple combination resulted in a stronger induction of glycolytic function, especially in control cells (Fig. 5B).

Conversely, analysis of oxygen consumption rate (OCR) revealed that mitochondrial respiration was significantly reduced in BE(2)-GR + ERα compared to control cells. Furthermore, activation of both receptors downregulated OXPHOS in BE(2)-GR + ERα. In contrast, neither E2, DEX, nor their combination affected mitochondrial activity in control cells (Fig. 5C). Notably, ATRA increased OXPHOS in both cell types whereas the mitochondrial parameters mildly decreased when combining E2 + DEX + ATRA, most likely due to a counter effect of E2 and DEX (Fig. 5B, D).

To investigate the metabolic potential in response to induced stress, we used oligomycin, inhibiting ATP synthase, and FCCP, an uncoupler of the electron transport chain. Upon stress induction, control cells shifted to a more energetic phenotype. BE(2)-GR + ERα cells moved towards an aerobic phenotype after ATRA treatment, with enhanced ECAR upon activation with ATRA or the triple cocktail (Fig. 5G and Supplementary Fig. 8C-F*,*). In BE(2)-GR + EV cells, ATRA alone or in combination with E2 + DEX increased glycolysis, whereas only a minor change was observed in OXPHOS (Fig. 5E, F). In contrast, in BE(2)-GR + ERα cells, E2 enhanced glycolysis and decreased respiration, an effect further potentiated when combining with DEX + ATRA for glycolysis while OXPHOS was unaffected compared to control. Notably, glycolysis was mildly enhanced with DEX but strikingly increased with ATRA but neither DEX nor ATRA influenced OXPHOS (Fig. 5G, H).

Lipids are stored in several cancer types as a consequence of metabolic reprogramming [49, 50]. We have previously demonstrated lipid droplet accumulation in ERα expressing NB cells upon E2-activation [17]. Hence, we explored lipid droplet formation upon simultaneous activation of GR, ERα, and RARα. Treatment with DEX, ATRA, their combination, or a mix of all three ligands resulted in lipid droplet accumulation in control cells. All treatments including E2 alone triggered lipid droplet deposition in BE(2)-GR + ERα cells while none of the vehicle conditions showed any sign of lipids (Fig. 5I).

Collectively, co-activation of GR, ERα, and RARα increased glycolysis and lipid droplet accumulation with minimal influence on mitochondrial respiration.

### Concurrent overexpression of GR and ERα reduced angiogenesis and tumor burden

**Fig. 6:**
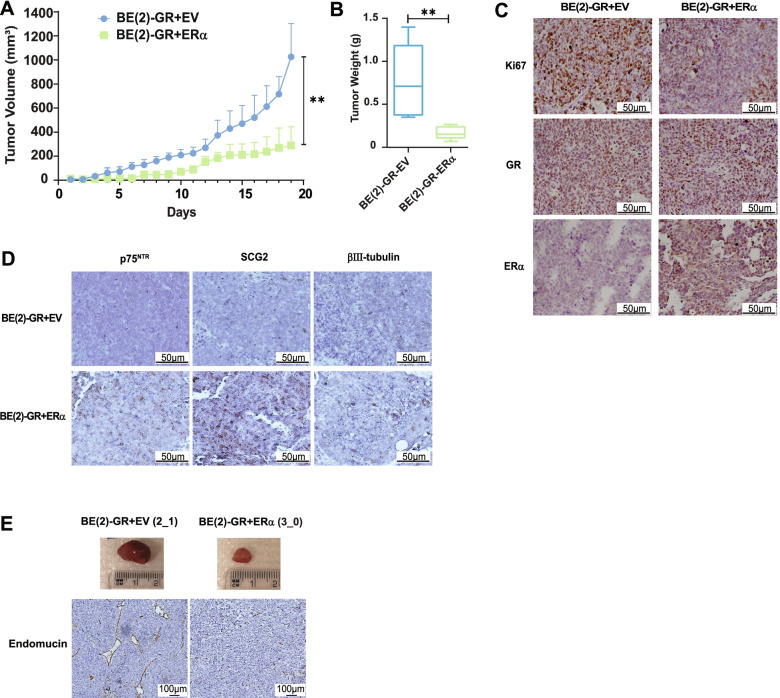
Combined GR and ERα-expression resulted in reduced angiogenesis and tumor burden. **A** Tumor volume in mice with tumors derived from BE(2)-GR + EV (*n* = 5) and BE(2)-GR + ERα (*n =* 6) cells. Growth was followed until the control group reached the ethical endpoint volume of 1 cm^3^. Data is presented as mean ± SD. Statistical analysis: Mann-Whitney *t*-test with * and ** indicating *p* < 0.05 and *p* < 0.01, respectively. **B** Tumor weight at experimental endpoint. Data is presented as mean ± SD. Statistical analysis: *t*-test with * and ** indicating *p* < 0.05 and *p* < 0.01, respectively. **C** Immunohistochemistry analysis of BE(2)-GR + EV and BE(2)-GR + ERα xenograft tumors stained with anti-Ki67, anti-GR, or anti-ERα antibodies. Scale bars indicate 50 μm. **D** Immunohistochemistry analysis of BE(2)-GR + EV and BE(2)-GR + ERα xenograft tumors stained with anti-p75^NTR^, anti-SCG2, or anti-βIII-tubulin antibodies. Scale bars indicate 50 μm. **E** Photos of tumors derived from BE(2)-GR + EV or BE(2)-GR + ERα cells with ruler. Images of immunohistochemistry analysis of the respective xenograft tumors stained with anti-endomucin for visualization of blood vessels. Scale bars indicate 100 μm. Representative images from at least five independent stainings per condition (**C**-**E**)

To evaluate the tumorigenic potential of combined expression of GR and ERα in vivo, cells were inoculated in nude mice, and tumor growth was followed until the control group reached the ethical endpoint volume. Overexpression of combined GR and ERα significantly reduced tumor burden and weight compared to tumors derived from GR expressing cells (Fig. 6A, B*,* and Supplementary Fig. 9A). We verified the presence of ERα and/or GR by analysis of tumor sections derived from control or BE(2)-GR + ERα cells. Ki67 staining showed that BE(2)-GR + ERα tumors had a less proliferative phenotype compared to controls (Fig. 6C). Staining of tumor sections showed higher levels of the differentiation markers, p75^NTR^, SCG2, and βIII-tubulin in tumors derived from BE(2)-GR + ERα cells compared to control tumors (Fig. 6D). Notably, tumors generated from BE(2)-GR + EV cells were more vascularized in appearance than those from BE(2)-GR + ERα cells. We confirmed extensive angiogenesis, with multiple and wide blood vessels, in BE(2)-GR + EV-derived tumors by staining for the endothelial marker endomucin in comparison with BE(2)-GR + ERα generated tumors (Fig. 6E and Supplementary Fig. 9B).

Together, these results demonstrated that co-activation of GR and ERα induced robust differentiation in combination with decreased angiogenesis and tumor burden in vivo, highlighting the potential of triggering both receptors as a putative therapeutic approach.

### The levels of* GR*, *ERα, and RARα* correlated with high expression of differentiation markers and favorable prognosis in NB patients

**Fig. 7:**
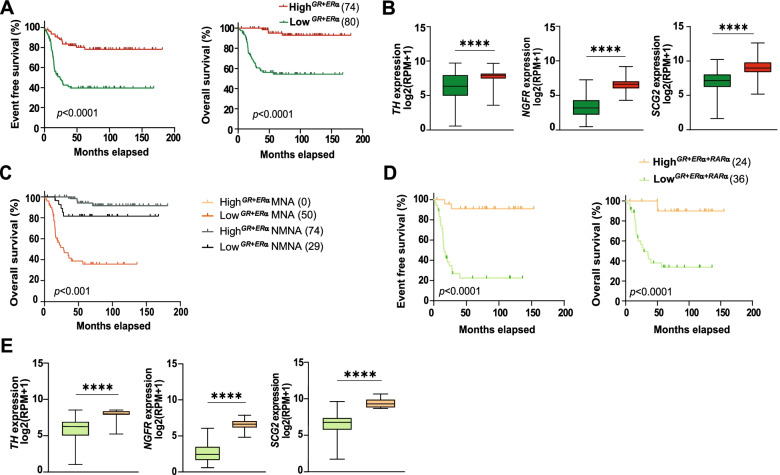
High *GR*, *ERα*, and *RARα* levels correlated with favorable prognosis and neuronal differentiation status in NB patients. **A** Kaplan-Meier overall and event free survival curves for patients divided into High^*GR + ERα*^ (74 patients) *versus* Low^*GR + ERα*^ (80 patients) mRNA expression levels. **B** mRNA expression of the neuronal differentiation markers *TH*, *NGFR*, and *SCG2* in the High^*GR* + *ERα*^ *versus* Low^*GR* + *ERα*^ patient groups. **C** Kaplan-Meier overall survival curves for patients divided into four patient groups according to *MYCN* status. MNA High^*GR + ER*^ (0 patients) *versus* Low^*GR + ERα + RARα*^ (50 patients) and NMNA with High^*GR + ER*^ (74 patients) *versus* Low^*GR + ERα + RARα*^ (29 patients) mRNA expression levels. **D** Kaplan-Meier overall and event free survival curves for patients divided into two groups according to High^*GR + ERα + RARα*^ (24 patients) *versus* Low^*GR + ERα + RARα*^ (36 patients) mRNA expression levels. **E** mRNA expression of the neuronal differentiation markers *TH*, *NGFR*, and *SCG2* between the High^*GR*+ *ERα + RARα*^ *versus *Low^*GR*+ *ERα + RARα*^ patient groups. Statistical analysis: *t*-test with *, ***, and **** indicating *p* < 0.05, *p* < 0.001, and *p* < 0.0001. Log-rank test was used for analysis of all Kaplan-Meier curves. *p*-values are shown in each plot. All data are from the SEQC cohort

Next, we interrogated the impact of the three receptors for survival of NB patients. Using the SEQC (*n* = 498) [27] and Kocak (*n* = 649) [28] NB cohorts, we analyzed the outcome of expression of *GR*, *ERα,* and *RARα,* either individually or in combination, for NB patient survival (Fig. 7). To this end, patients were divided in high or low *GR*, *ERα,* or *RARα*, according to their mRNA levels and separated in quartiles of expression: from quartile 1, patients with the highest levels, to quartile 4, patients with the lowest expression. We selected all patients in quartile 1 (High^*GR*^, High^*ERα*^, or High^*RARα*^) and in quartile 4 (Low^*GR*^, Low^*ERα*^, or Low^*RARα*^), separating them from patients with intermediate expression levels (quartiles 2 and 3). Patients with higher *GR*, *ERα,* or *RARα* mRNA levels had a better event free and overall survival than patients with lower levels in the SEQC cohort (Supplementary Fig. 10A-C). However, only patients with High^*GR*^ mRNA expression correlated with a better overall survival in the Kocak dataset (Supplementary Fig. 10D-F). We therefore also analyzed the Oberthuer cohort (*n* = 251) [29], where patients with high levels of all the individual receptors presented with better prognosis (Supplementary Fig. 10G-I), in agreement with our previous data [16–18]. Additionally, since we already had demonstrated a negative correlation between MYCN and the three NHRs, we further studied the levels of *GR*, *ERα*, and *RARα* in the SEQC dataset separating the patients according to their *MYCN* status, either *MYCN*-amplified (MNA) or non-*MYCN*-amplified (NMNA). As expected, patients with *MYCN*-amplification showed lower levels of all three receptors than those lacking amplification (Supplementary Fig. 10 J).

Given these results, we investigated the clinical relevance of concurrent overexpression of GR and ERα. The SEQC and Kocak datasets were divided into patients with High^*GR + ERα*^ and Low^*GR + ERα*^ mRNA levels. As expected, the high co-expression patient group was related to favorable prognosis with better event free and overall survival compared to the low expression group in both cohorts (Fig. 7A and Supplementary Fig. 11A).

As our in vitro and in vivo experiments showed strong induction of neuronal differentiation by GR and ERα co-expression, we compared levels of differentiation markers between the High^*GR + ERα*^ *versus* Low^*GR + ERα*^ patient groups. Notably, expression of *NGFR* (*p75*^*NTR*^), *TH*, and *SCG2*, were significantly higher in High^*GR + ERα*^ patients in contrast to those with lower *GR* and *ERα* levels (Fig. 7B and Supplementary Fig. 11B). We next divided patients with High^*GR + ERα*^ and Low^*GR + ERα*^ levels according to *MYCN* status using the SEQC dataset. Strikingly, no single patient with *MYCN*-amplification and High^*GR + ERα*^ was identified, further validating the role of MYCN in regulating NHRs expression. As expected, High^*GR + ERα*^ NMNA patients had a better survival than the Low^*GR + ERα*^ NMNA patients (Fig. 7C).

Using Gene Set Enrichment Analysis (GSEA), we identified processes with significant differences between the High^*GR + ERα*^ *versus *Low^*GR + ERα*^ patient groups (see Additional Files 1 and 2 containing the gene sets used for the analysis), including neuronal crest differentiation, regulation of actin cytoskeleton, early differentiation genes, and axon guidance. Moreover, we also found metabolism-related processes including fatty acid biosynthesis, and fatty acid β-oxidation although they did not reach the significant threshold (Supplementary Fig. 11C).

We anticipated that patients with high expression of the three receptors were associated with elevated levels of differentiation markers and a better prognosis than patients with high levels of two NHRs. Despite the relatively low number of patients in the High^*GR + ERα + RARα*^ (*n *= 24 in SEQC and *n = *nine in Kocak) *versus* Low^*GR + ERα + RARα*^ (*n *= 36 in SEQC and *n *= eight in Kocak) mRNA expression groups, we observed very large differences in survival between these quartiles in both cohorts (Fig. 7D and Supplementary Fig. 11D), although for Kocak patients, differences were not statistically significant. Accordingly, neuronal differentiation markers showed higher expression in High^*GR + ERα + RARα*^ patients *versus* the Low^*GR + ERα + RARα*^ patient group in both datasets (Fig. 7E and Supplementary Fig. 11E).

Collectively, in silico analyses demonstrated that combined high levels of *GR*, *ERα*, and *RARα* correlated with increased expression of neuronal markers and a more favorable outcome linked to a more differentiated state in tumors of NB patients.

### Single-nuclei transcriptome analysis suggested subsequent *GR*, *ERα*, and *RARα* expression for signaling a transition from undifferentiated to adrenergic cells

To explore the physiological significance of *GR*, *ERα*, and *RARα* during development of the human sympathetic nervous system, we studied their expression in four embryonic and seven adult adrenal glands, using the Suntsova dataset [30]. Expression of some of the genes of interest were available, namely the genes encoding GR (*NR3C1*), p75^NTR^ (*NGFR*), βIII-tubulin (*TUBB3*), and the Erb-B2 Receptor Tyrosine Kinase 3 (*ERBB3*). Of these, only *GR* showed differences, with higher expression in embryonic adrenal glands compared to adult (Supplementary Fig. 12A).

**Fig. 8:**
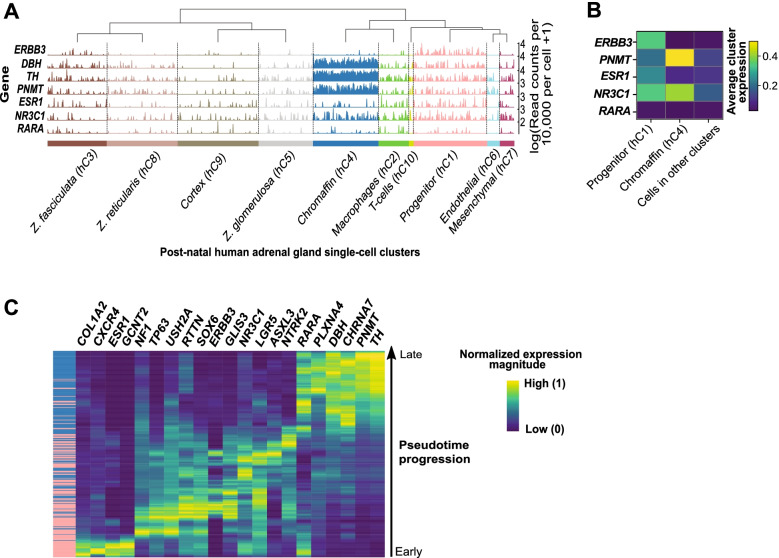
The three NHRs *ERα*, *GR,* and *RARα* were sequentially expressed during human adrenal gland development. **A** Tracksplot illustrating expression of the genes encoding ERα (*ESR1*), GR (*NR3C1*), and RARα (*RARA*) in ten different cell populations from three normal human post-natal adrenal glands (31). These include cortex cells *i.e*.*,* Zona fasciculata (hC3), Zona reticularis (hC8), Zona glomerulosa (hC5), and cortex (hC9), immune cells [macrophages (hC2), and T-cells (hC10)], endothelial (hC6), mesenchymal (hC7), and progenitor cells (hC1). Reference genes were *ERBB3* for progenitors, *DBH* and *TH* for noradrenergic, and *PNMT* for adrenergic cells. Gene expression was computed as log (read counts per 10,000 per cell + 1). One-tailed Welch’s *t*-test was used with FDR < 0.01. **B** Matrix plot illustrating the average expression of *ESR1*, *NR3C1*, and *RARA*, and reference genes for progenitor (*ERBB3*) and chromaffin (*PNMT*) cells. Significance was tested with one-tailed Welch’s *t*-tests and a FDR threshold of 0.01. Gene expression was computed as log (read counts per 10,000 per cell + 1). The top average expression displayed was limited to 0.45 (for *PNMT* with an average value of 1.34 in chromaffin cells). **C** Pseudotime reconstruction of differentiating chromaffin cells in human adrenal glands. The population of progenitor cells in post-natal adrenal gland (pink) sourcing from chromaffin cells (blue), were separated from early to late (left panel). The reconstruction of this process indicated that gene expression elapses from an undifferentiated stem-like (* i.e., RTTN+*) to an adrenergic signature (*TH+, PNMT+*). **B-C** Normalized expression magnitude from Low (0; dark blue) to High (1; yellow) as indicated to the right

As a proxy to study neural differentiation in vitro and to overcome its limitations, we interrogated the role of *GR*, *ERα*, and *RARα* during chromaffin cell differentiation in single-cell-nuclei of embryonic and post-natal human adrenal glands [28, 29]. Expression was compared with reference genes including *ERBB3* for progenitor cells, *Dopamine Beta-Hydroxylase* (*DBH*) as well as *TH* for nor-adrenergic population, and phenylethanolamine N-methyltransferase (*PNMT*) for adrenergic cells. We identified a significant expression of *ERα* (*ESR1*) in the progenitor population of chromaffin cells in post-natal adrenal gland (hC1, FDR = 0.008, one-tailed Welch’s *t*-test) (Fig. 8A). We found that *GR* (*NR3C1*) was significantly upregulated in chromaffin cells (hC4, FDR = 5.18 × 10-4, one-tailed Welch’s *t*-test) and their progenitor cluster (hC1, FDR = 2.82 × 10-3, one-tailed Welch’s *t*-test) in post-natal adrenal gland in accordance with the Suntsova dataset analysis (Supplementary Fig. 12A). In contrast, *RARα* was not significantly expressed in any post-natal human adrenal gland cell cluster (FDR > 0.01 for all clusters, one-tailed Welch’s *t*-test) (Fig. 8A).

We then examined the expression of *GR*, *ERα*, and *RARα* in the developing human adrenal gland using previously published data [32]. Following the corrected annotation [31, 51], a significant up-regulation of *GR* in chromaffin cells and of *RARα* in non-cycling chromaffin cells was reported (Fig. 8B).

Next, we generated a pseudotime reconstruction of differentiating chromaffin cells in post-natal human adrenal glands to identify the order of expression of the three *NHR* genes during neuronal development. Progenitor cells were separated from early to late cells, the former with expression of progenitor markers and a high differentiation potential, and the latter sourcing from chromaffin cells, characterized by nor- and adrenergic markers and a lower differentiation capacity. This trajectory showed that *ESR1*, *NR3C1*, and *RARA* were sequentially expressed during chromaffin development (Fig. 8C).

In conclusion, our data suggest that the three NHR genes, *GR*, *ERα,* and *RARα*, are sequentially expressed from progenitor to chromaffin cells during human adrenal gland differentiation, indicating important roles during maturation of the sympathetic nervous system.

## Discussion

The current standard treatment for high-risk NB includes surgery, chemotherapy, myeloablative therapy followed by autologous hematopoietic stem cell transplantation. In addition, maintenance therapy with 13-*cis*-RA (isotretinoin) with or without anti-disialoganglioside 2 (GD2)-antibodies is used in some European countries and in the United States [15, 36]. Retinoids are important factors for cellular homeostasis of many normal adult and embryonic tissues, with potent antiproliferative activity against several tumors [52]. Combination therapies may enhance the anticancer efficacy of retinoids [53]. For instance, a cocktail of retinoids and histone deacetylase inhibitors showed a synergistic effect in NB [54] and treatment with ceramide modulators increased the anti-tumor potential of RA in NB models [6]. Isotretinoin has undergone several clinical trials for NB treatment, both alone or in combination with chemotherapy, transplantation, or immunotherapy. Whereas RA alone did not improve event-free survival, it provided a benefit when used in combination with other approaches [55, 56]. It is important to highlight that none of the studies with RA used patients stratified according to RARα levels. Despite advancements, half of all patients treated with 13-*cis*-RA still relapse, showing the need for a potent combination therapy approach for high-risk NB.

In this study, our aim was to analyze the combined activation of GR, ERα, and RARα, with dexamethasone (DEX), 17-β estradiol (E2), and all-*trans* retinoic acid (ATRA), on the differentiation phenotype of NB cells. Importantly, the concentration of all ligands used was much lower than their respective IC_50_. Using in vitro, in vivo*,* and in silico approaches we concluded that the robust effects achieved upon triple treatment support the potential of translating this strategy into clinical practice. A combination of DEX, E2, and ATRA may provide a benefit for NB patients with high levels of the three receptors.

Dexamethasone (DEX) is commonly used together with chemotherapy against certain types of cancer [57–59] as well as for treating edema in children [60]. Previously, we showed that GR activation by glucocorticoids drives sympathetic neuronal differentiation in mice. Upon *MYCN*-overexpression, neuroblasts/neurons that would otherwise express GR, failed to initiate or maintain their differentiation status and instead developed proliferative behavior. In support, hyperplasic lesions in ganglia of *TH*-*MYCN* mice expressing MYCN were deficient in GR and had low levels of neuronal differentiation markers [18].

Our previous studies further showed that MYCN induced *miR-18a*, one member of the *17 ~ 92* miRNA cluster, which in turn downregulated ERα, thereby interfering with E2-stimulated neuronal differentiation. Thus, the consequence of E2 signaling in NB is the opposite to the well-known oncogenic effect in breast cancer [61, 62]. In NB, ERα overexpression is partly sufficient to overcome the malignant phenotype associated with MYCN overexpression supported by increased levels of differentiation markers [17].

Here, we further explored the differentiation phenotype induced by DEX treatment in BE(2) cells overexpressing GR, as well as validated our findings in two non-*MYCN*-amplified cell lines overexpressing GR, SK-N-AS and SH-SY5Y. Exposure of SK-N-AS-GR cells with DEX, ATRA and the combination resulted in glial differentiation while as expected SH-SY5Y-GR cells displayed a stronger neural differentiation upon ATRA or DEX + ATRA than to DEX alone.

To investigate whether the observed effects on viability, differentiation, and tumorigenesis could be potentiated by two NHRs, we transduced the BE(2)-GR overexpressing cells with *ERα*, and extended our study to two additional *MYCN*-amplified NB cell lines, IMR32 and KCN69n. Upon E2 + DEX treatment, the differentiated phenotype was more robust in cells expressing both GR and ERα in comparison with cells with single receptors. The triple ligand cocktail further enhanced the levels of neural differentiation markers and generated a more interconnected network of neurites compared to individual or double treatments in the BE(2)-, IMR32-, and KCN-GR + ERα cells. As expected, we also observed changes in MYCN levels. Generally, a decrease in MYCN is observed upon differentiation-inducing treatments in the cell lines used in the study. However, we observed a slight increase in MYCN upon treatment in some of replicates of BE(2) cells. This upregulation could be due to the requirement of MYCN expression for the proliferative burst at the onset of the differentiation program [63]. Moreover, we observed an increase in viability upon low concentrations of ATRA treatment in the GR-overexpressing and GR + ERα-overexpressing cells. When combining ATRA with E2 and DEX, the viability was significantly decreased, relative to the control levels. Further analysis of these differences in viability are needed in future studies.

Recently, Siaw et al., described *DLG2* as a possible tumor suppressor gene in NB since tumors with 11q deletion present genetic lesions in the *DLG2* gene, and its downregulation is associated with a reduction in the neural differentiation induced by ATRA [44]. Interestingly, we observed enhanced *DLG2* expression levels in all differentiation inducing conditions both in BE(2)-GR + EV and BE(2)-GR + ERα cells, indicating that DLG2 is important also for the neuronal phenotype provoked by the activation of GR and ERα.

Importantly, our in vitro data was validated in vivo*,* showing a robust augmentation in the levels of neural differentiation markers with a decrease in volume and weight, and with a less angiogenic phenotype in tumors simultaneously overexpressing GR and ERα compared to those generated from control cells. This is in line with the report that ERα overexpression inhibited angiogenesis in a model of human endometrial cancer [64]. Our data suggest that ERα activation is causing reduced tumor vascularization, as we did not see a similar phenotype in tumors from GR-overexpressing cells.

During recent years, it has been shown that many tumors can oxidize glucose via OXPHOS, contributing to cancer progression and aggressiveness [65, 66]. Here, we demonstrate that activation of GR and ERα shifted metabolism to glycolysis, which substantially increased upon treatment with ATRA or the triple combination. Although the three ligands induced robust neuronal differentiation, E2 and DEX affected mitochondrial function in an opposite manner compared to ATRA, indicating that a specific shift in OXPHOS is not a general characteristic of neuronal differentiation. Another interpretation yet unexplored, is that activation of RARα induces differentiation faster than stimulation of GR and ERα, and that OXPHOS might decrease after a longer incubation. We uncovered that BE(2)-GR + EV cells have a more energetic phenotype compared to BE(2)-GR + ERα cells after inducing metabolic stress. When activating BE(2)-GR + ERα with E2 + DEX, cells shifted towards glycolysis. Most likely, this could be an adaptation to the reduced mitochondrial activity, as ATP production is diminished after coactivation of both NHRs.

Alterations in energy production, as those reflected by the reprogramming observed, could impact the complex regulation of lipid metabolism and the formation of lipid droplets. These are neutral lipid storage organelles that regulate lipid homeostasis in the cells according to their nutrient requirements. They promote cancer survival by controlling the levels of polyunsaturated fatty acids (PUFAs) and lipotoxicity, which otherwise would result in increased oxidative stress and cell death [67, 68], as well as by providing resistance to common chemotherapeutic drugs [69]. We have previously demonstrated that *MYCN*-amplified NB cells depend on lipid metabolism, since targeting both fatty acid β-oxidation as well as de novo fatty acid synthesis resulted in reduction of tumor burden and the latter also induced neuronal differentiation [70, 71]. Upon ATRA treatment, OXPHOS parameters were slightly elevated, together with a robust increase in the glycolytic function. This indicated a shift towards a glycolytic phenotype with less mitochondrial usage, which could explain the reduction in β-oxidation and in turn, lipid accumulation. This is in line with our previous results showing lipid deposits after MYCN inhibition as a consequence of a diminished fatty acid β-oxidation [72]. In addition, when we treated ERα-overexpressing NB cells with E2 and NGF, lipid droplet accumulation occurred due to impairment of fatty acid utilization [17]. Thus, exploring the role of lipid droplets in the metabolic reprogramming taking place during differentiation could provide new insights about their potential as targets in NB.

The GSEA analysis revealed differences in processes related to neural differentiation as well as metabolic processes including fatty acid metabolism in agreement with lipid droplet accumulation. When examining patients in the High^*GR + ERα + RARα*^ *versus *Low^*GR + ERα + RARα*^ mRNA expression groups, a strikingly stronger difference in survival curves and a more robust upregulation of neuronal differentiation markers compared with patients with double receptor expression was observed. Importantly, expression of the *NHR* genes negatively correlated with *MYCN*-amplification, as non-*MYCN*-amplified cell lines showed higher levels of the genes encoding these three receptors compared to *MYCN*-amplified tumors. Remarkably, no patients with *MYCN*-amplification and High^*GR + ERα*^ levels were identified supporting our hypothesis.

The glucocorticoid receptor (GR) is implicated in cell growth, proliferation, and neuronal plasticity during development of the nervous system [73, 74]. In the sympathetic nervous system, this NHR is mainly elevated in ganglia and specifically in chromaffin cells, inducing differentiation of catecholaminergic cells [75]. We have previously shown that nuclear GR was present in postnatal sympathetic ganglia in wild-type mice while absent in MYCN-positive hyperplastic areas or tumors from *TH-MYCN* mice [18]. Our present data showed that *GR (NR3C1)* was present both in human progenitor and chromaffin cells and was highly expressed in fetal adrenal glands, in concert with a recent report showing *GR* expression in human chromaffin cells [76]. This indicates that GR is important both at early stages and for terminal differentiation of sympathetic neurons. The mRNA levels of *ERα* (*ESR1*) were significantly elevated in progenitor cells, in agreement with our previous data demonstrating ERα expression in human fetal sympathetic ganglia [16]. Another study reported significant *ESR1* expression in human fetal adrenal cortex and in mesenchymal NB cells. These authors also observed significant *NR3C1* and *RARA* expression in several neural populations and vascular cells of human fetal adrenal gland as well as in mesenchymal cells of NB [51]. Together, these data postulate an essential role of *ERα* during sympathetic nervous system maturation. ATRA together with bone morphogenic proteins (BMPs) was shown to regulate expression of the *trkA* and *trkC* neurotrophin receptor genes in developing sympathetic neurons [77]. Additionally, retinoids coordinated polarity and cell shape in sympathetic ganglia during development [78]. Our analysis of ten different cell populations from postnatal human adrenal glands demonstrated that although *RARA* expression was not significantly upregulated in chromaffin cells, levels were high in non-cycling chromaffin cells in a statistically significant manner [32]. One possibility is that *RARA* is only expressed during development of the nervous system although we cannot exclude that the restricted sample size of postnatal glands (*n* = 3), limits the significance of *RARA* expression.

Notably, *GR* has been characterized as part of the Group 2 neural crest cell (NCC)/core regulatory circuitries (CRCs)/mesenchymal signature in NB cells [79, 80]. One possibility is that *GR*, *ERα*, and *RARA* act in concert to promote differentiation from undifferentiated to noradrenergic cells. Expression of *ERα* in progenitor and undifferentiated cells could initiate differentiation, continued by *GR* expression in transitioning cells, and ending with *RARA* in more differentiated cells. In agreement with this scenario, a pseudotime reconstruction from progenitor to chromaffin cells in the adrenal gland indeed suggested sequential expression of *ERα*, *GR*, and *RARα*, following differentiation of progenitor cells. Particularly, cells initiated expressing some mesenchymal and progenitor genes (*e.g*., *COL1A2*, *NF1*, *RTTN*, *ERBB3*) to transition into the expression of noradrenergic and adrenergic markers (*e.g*., *DBH*, *TH*, *PNMT*). The expression of *ESR1* was found within the first, and *RARA* within the latter groups of genes, while *NR3C1* was placed at the interface. The dynamics of the individual gene expression pattern indicate that in some cases, cells placed at different positions in the pseudotime can express the same gene, possibly as a consequence of stochastic expression or misplacement in the trajectory. This is for instance the case for *RARA*, which is expressed both in early and late stages. Although this can suggest a role of *RARA* in the early stages of the pseudotime, most of the progenitor cells with *RARA* expression are located later in proximity of *RARA*-expressing chromaffin cells. Nevertheless, we cannot discard the option that the sequential gene expression could signal a transdifferentiation cue for other types of cells, including neuroblast/sympathoblast cells, especially while they retain plasticity during development [32, 76].

## Conclusions

**Fig. 9:**
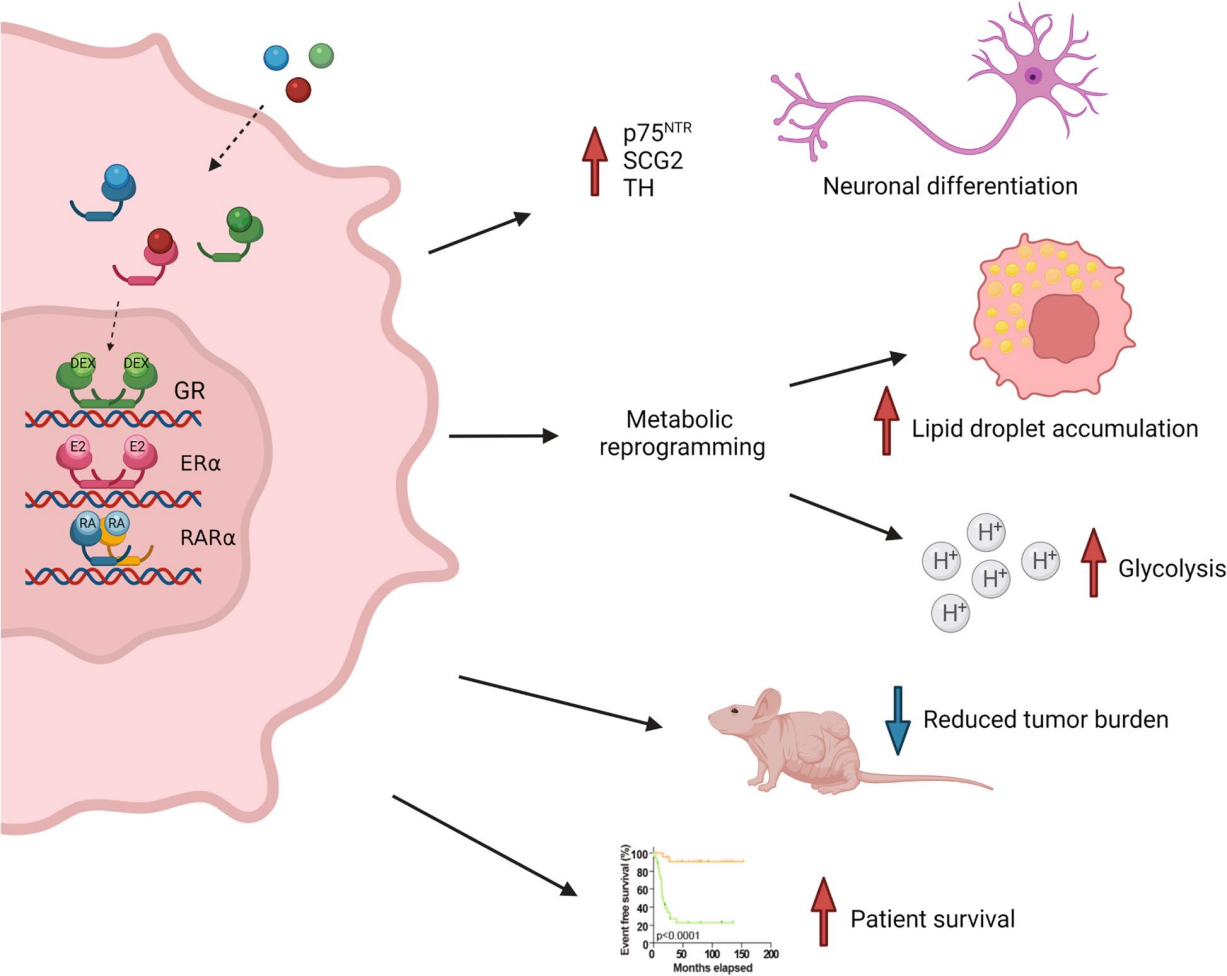
Graphical abstract. Activation of the three NHRs via E2, DEX, and ATRA results in profound changes in NB cells with combined ERα and GR overexpression. Cells respond by morphological changes and neurite outgrowth with upregulation of the p75^NTR^, SCG2, and TH neuronal differentiation markers. The three ligands also induce metabolic reprogramming manifested by increased glycolysis and accumulation of lipid droplets. Mice with xenografts from GR- and ERα-overexpressing neuroblastoma cells, where NHR signaling is activated by endogenous mouse ligands, show reduced tumor growth compared to control. Importantly, neuroblastoma patients with high levels of *ERα*, *GR*, and *RARα* show favorable prognosis and survival while patients with low levels of these three receptors show a poor outcome. The figure was created with Biorender.com

Collectively, our findings show that concurrent stimulation of GR, ERα, and RARα strongly potentiates neuronal differentiation, triggers metabolic reprogramming, and reduces tumor burden (Fig. 9). Single-nuclei RNA sequencing analysis revealed that these receptors are sequentially expressed during the differentiation of chromaffin cells in the post-natal human adrenal gland. Importantly, combined high expression of the genes encoding the three receptors correlates with a favorable prognosis. Together, these data set the basis for investigation of the therapeutic potential of NHR activation as a strategy to induce differentiation for high-risk NB therapy.

## Supplementary Information

**Additional file 1.**

**Additional file 2.**

**Additional file 3.**

## Data Availability

All data generated and/or analyzed during the current study are available from the corresponding author on reasonable request. The patient datasets analyzed during the current study (SEQC, GSE62564; Kocak, GSE45547; Suntosva, GSE96631), are available in the GEO repository (https://www.ncbi.nlm.nih.gov/geo/), and Oberthuer (E-MTAB-38) dataset in the ArrayExpress platform (https://www.ebi.ac.uk/arrayexpress/). The single-cell nuclei transcriptomic dataset was obtained from Supplementary Dataset 2 in Bedoya-Reina et al. 2021 and Dong et al. 2020.

## Abbreviations

ATRA: All-*Trans*-Retinoic Acid
B2M: β-2-Microglubulin
BMP: Bone morphogenetic Protein
CRCs: Core Regulatory Circuits
DBH: Dopamine Beta-Hydroxylase
DEX: Dexamethasone
DLG2: Discs Large MAGUK Scaffold Protein 2
E2: 17-β-Estradiol
ECAR: Extracellular Acidification Rate
ERBB3: Erb-B2 Receptor Tyrosine Kinase 3
ERα: Estrogen Receptor α
FCCP: Carbonyl Cyanide 4-(trifluoromethoxy) Phenylhydrazone
GD2: disialoganglioside 2
GFAP: Glial Fibrillary Acidic Protein
GR: Glucocorticoid Receptor
GSEA: Gene Set Enrichment Analysis
INSS: International Neuroblastoma Staging System
NB: Neuroblastoma
NCC: Neural Crest Cells
NEFL: Neurofilament light chain
NGF: Nerve Growth Factor
NGFR: Nerve Growth Factor Receptor
NHR: Nuclear Hormone Receptor
OCR: Oxygen Consumption Rate
OXPHOS: Oxidative Phosphorylation
p75^NTR^: Neurotrophin Receptor p75
PNMT: Phenylethanolamine N-methyltransferase
PUFA: Polyunsaturated fatty acid
RA: Retinoic Acid
RARs: Retinoic Acid Receptors
RARα: Retinoic Acid Receptor *α*
SCG2: Secretogranin-2
SOX2: Sex determining Region Y-box 2
TH: Tyrosine Hydroxylase
TRKA/TRKC: Tropomyosin Receptor Kinase A/C
TUBB3: βIII-tubulin
VIM: Vimentin
