# Supplementary material for: Expression and activation of nuclear hormone receptors result in neuronal differentiation and favorable prognosis in neuroblastoma
Source: J Exp Clin Cancer Res. 2022 Jul 19;41:226. doi: 10.1186/s13046-022-02399-x (PMC9295514; doi:10.1186/s13046-022-02399-x)
Supplement: Supplementary file 3 — Additional file 3. [file 13046_2022_2399_MOESM3_ESM.pdf]

Supplementary Information for

Expression and activation of nuclear hormone receptors result in neuronal differentiation and favorable prognosis in neuroblastoma.

Lourdes Sainero-Alcolado[a], Muhammad Mushtaq[a,b,*], Judit Liaño-Pons[a,*], Aida Rodriguez-Garcia[a], Ye Yuan[a], Tong Liu[a,c], María Victoria Ruiz-Pérez[a], Susanne Schlisio[a], Oscar Bedoya-Reina[a], and Marie Arsenian-Henriksson[a,1]

[a]Department of Microbiology, Tumor and Cell Biology (MTC), Biomedicum B7, Karolinska Institutet, SE-171 65, Stockholm, Sweden.

[b]Present address: Department of Biotechnology, Faculty of Life Sciences and Informatics, Balochistan University of Information Technology, Engineering, and Management Sciences, 87300 Quetta, Pakistan.

[c]Present address: Department of Medicine, Center for Molecular Medicine (CMM), Karolinska Institutet, SE-171 64 Stockholm, Sweden.

*Equal contribution.

[1]To whom correspondence should be sent:
Marie Arsenian-Henriksson
Email: marie.arsenian.henriksson@ki.se.

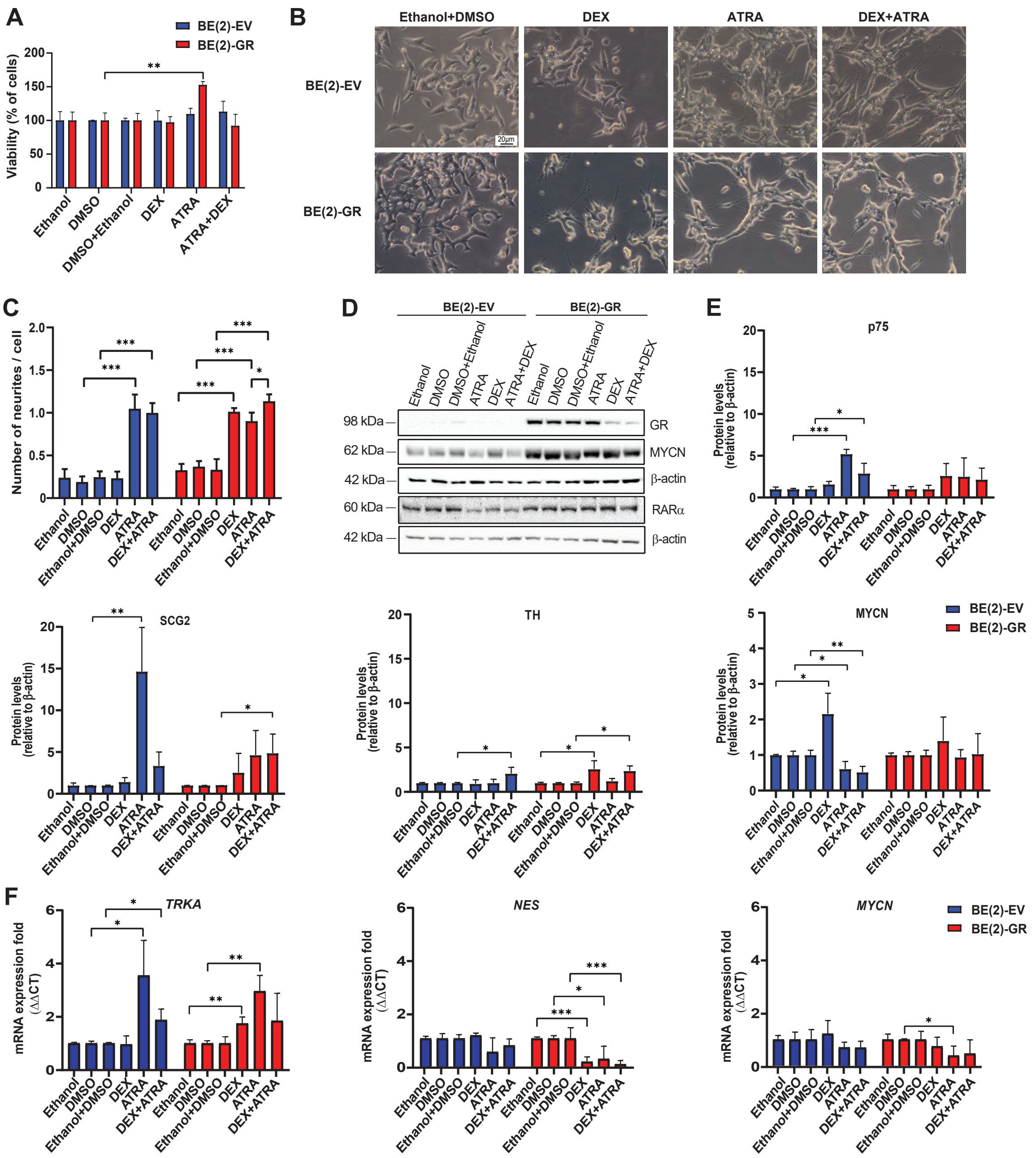

Supplementary Fig. 1 related to Figure 1.

A) Percentage of viable cells determined by WST-1 after activation of GR. Cells were treated with 100 nM DEX, 0.5 µM ATRA, or their combination for three days. All conditions were normalized to the respective control (100 % viability).

B) Neurite outgrowth assay after seven days treatment of BE(2)-EV and BE(2)-GR with 100 nM DEX, 0.5 µM ATRA, or their combination. Representative phase contrast microscopy images from three independent experiments. Scale bars represent 20 µm.

C) Quantification of neurites in the experiment shown in **B**. Data is presented as number of neurites per cell in three different images from three independent experiments.

D) Western blot of the indicated proteins in BE(2)-EV and BE(2)-GR cells following treatment with 100 nM DEX, 0.5 μM ATRA, or their combination during seven days. Proteins were separated on two gels with β-actin as loading control. Molecular weight markers shown to the left. Representative blots from three independent experiments.

E) Densiometric analysis of Western blots from three independent experiments of the indicated proteins from BE(2)-EV and BE(2)-GR represented in **D** (MYCN) and **Figure 1B**.

F) mRNA expression levels of the indicated genes in BE(2)-EV and BE(2)-GR cells following treatment with 100 nM DEX, 0.5 μM ATRA, or their combination during seven days. *B2-microglobulin* (*B2M*) and *β-actin* were used as control genes.

Data in **A** and **C, E,** and **F** are presented as mean ± SD of at least three independent experiments; statistical analysis: *t*-test, with *, **, ***, and **** indicating $p < 0.05$, $p < 0.01$, $p < 0.001$, and $p < 0.0001$.

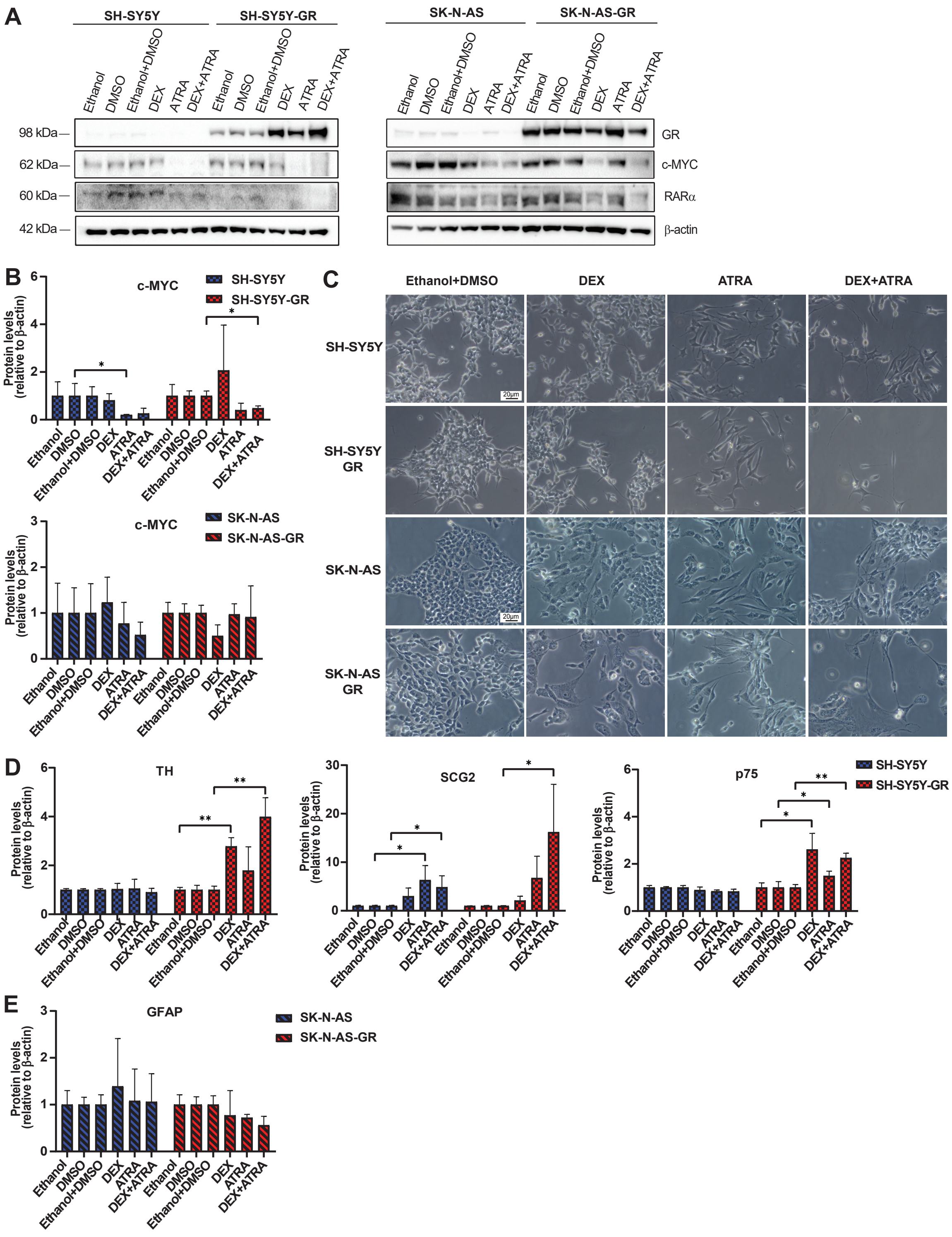

Supplementary Figure 2 related to Figure 1.

A) Western blot of indicated proteins in parental SH-SY5Y, SH-SY5Y-GR, parental SK-N-AS, and SK-N-AS-GR cells following treatment with 100 nM DEX, 5 μM ATRA, or their combination during seven days. Proteins were separated on two gels with β-actin as

loading control. Molecular weight markers shown to the left. Representative blots from three independent experiments.

B) Densiometric analysis of c-MYC protein from parental SH-SY5Y and SH-SY5Y-GR as well as from parental SK-N-AS and SK-N-AS-GR from the Western blots represented in **A**.

C) Neurite outgrowth assay after seven days treatment with 100 nM DEX, 5 μM ATRA, or their combination. Representative phase contrast microscopy images from three independent experiments. Scale bars indicate 20 μm.

D) Densiometric analysis of the indicated proteins from parental SH-SY5Y and SH-SY5Y-GR from the Western blots represented in **Figure 1E**.

E) Densiometric analysis of the indicated protein from parental SK-N-AS and SK-N-AS-GR from the Western blots represented in **Figure 1G**.

The densitometric analyses in **B**, **D**, and **E** were performed from Western blots from three independent experiments and are shown as mean ± SD; statistical analysis: *t*-test, with *, **, indicating $p < 0.05$ and $p < 0.01$, respectively.

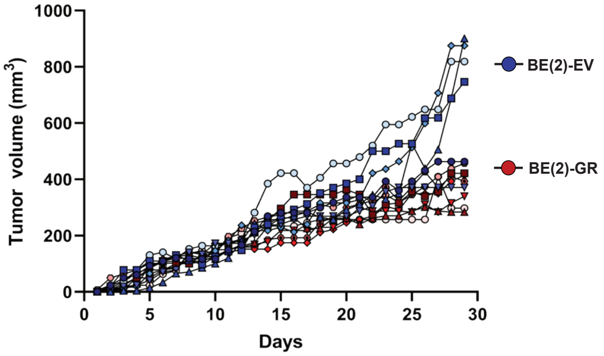

Supplementary Figure 3 related to Figure 2.

Tumor volume for each mouse in the xenograft experiment comparing tumors from BE(2)-EV (blue) and BE(2)-GR (red) cells, respectively; *n*= 6 in the BE(2)-EV group and *n*= 7 in the BE(2)-GR group. Tumor growth was followed until the control group reached the ethical endpoint volume of 1 cm^3. The summary of the experiment is shown in **Figure 2A**.

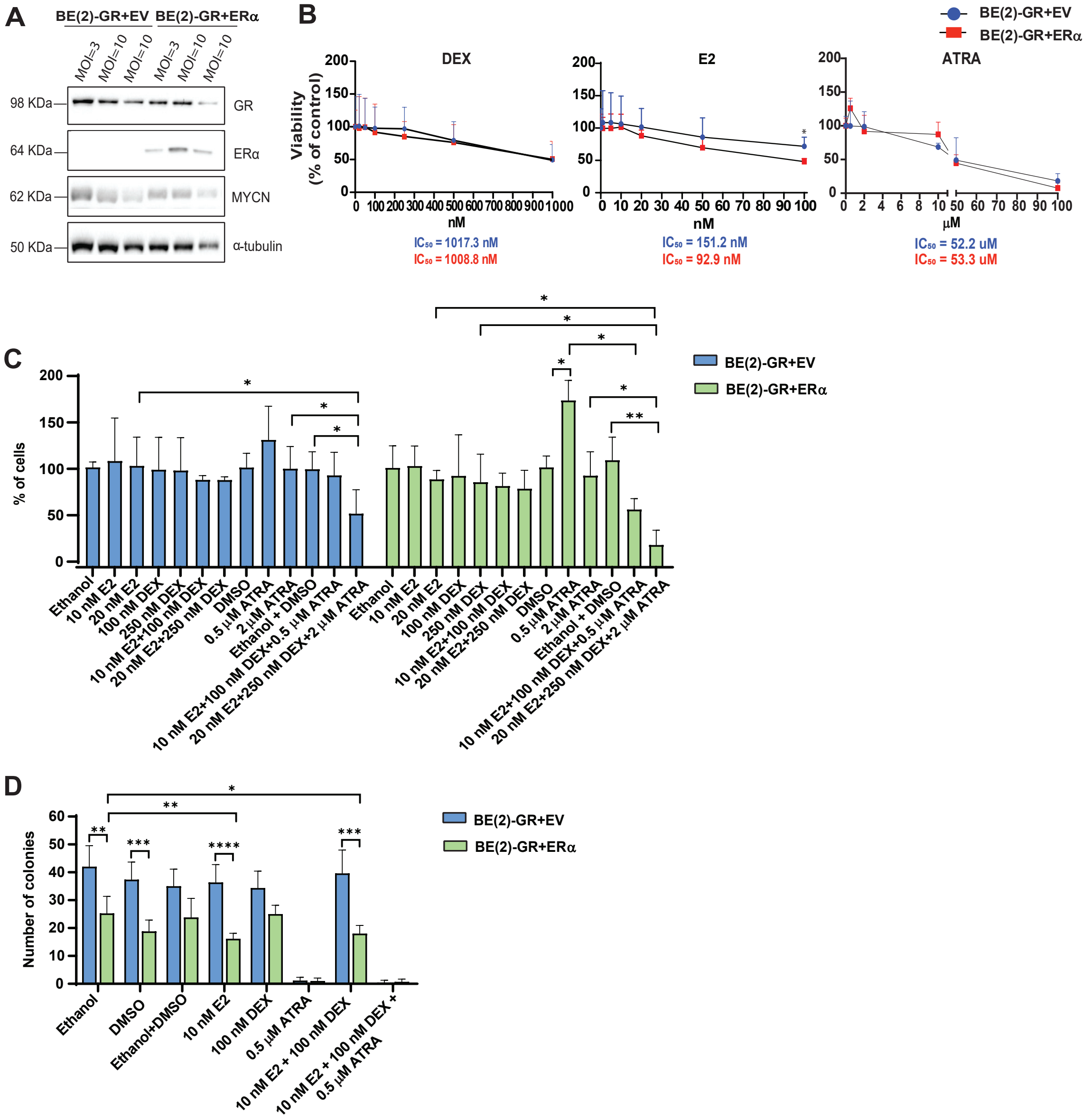

Supplementary Figure 4 related to Figure 3.

A) Western blot analysis of GR, ERα, and MYCN in BE(2)-GR+EV and BE(2)-GR+ERα cells at different multiplicity of infection (MOI) as indicated. α-tubulin was used as loading control. Molecular weight markers are shown to the left. Blots are representative of three independent experiments.

B) Viability graphs for IC_{50} calculation of DEX, E2, and ATRA after 72 h of treatment with the indicated concentrations in BE(2)-GR+EV (blue) and BE(2)-GR+ERα (red) cells.

C) Percentage of viable cells determined by WST-1 after activation of GR and ERα in BE(2)-GR+EV (blue) and BE(2)-GR+ERα (green) cells. Viability is presented as 100 % compared to control levels. Cells were treated with E2, DEX, ATRA, or in combination as indicated for three days.

D) Colony numbers obtained from BE(2)-GR+EV (blue) and BE(2)-GR+ERα (green) cells after ten days of treatment with ligands as indicated. ImageJ software was used for quantification.

Data in **B**-**D** are presented as mean ± SD of at least three independent experiments; statistical analysis: *t*-test, with *, **, ***, and **** indicating $p < 0.05$, $p < 0.01$, $p < 0.001$, and $p < 0.0001$.

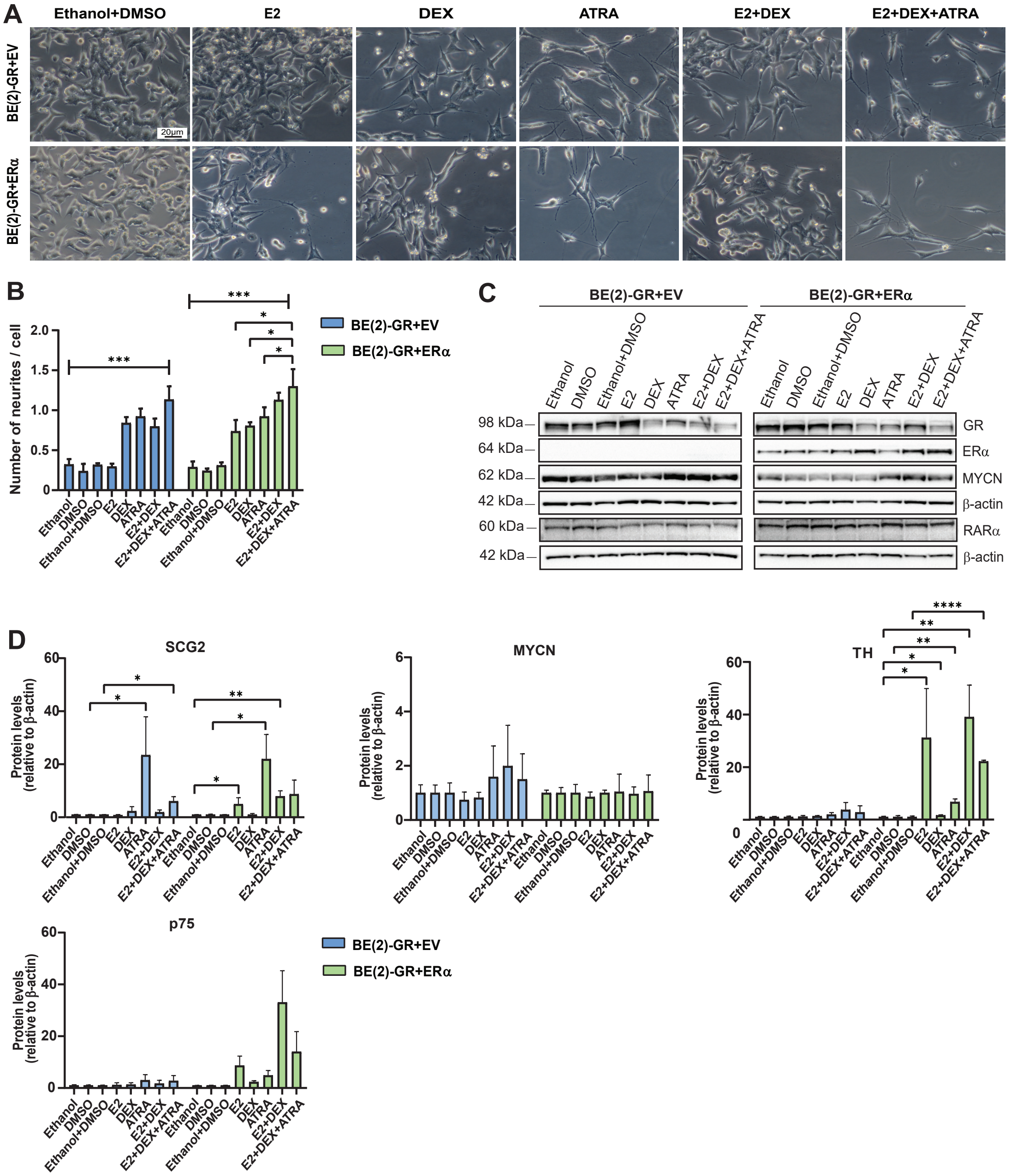

Supplementary Figure 5 related to Figure 3.

A) Neurite outgrowth assay after seven days of treatment with 10 nM E2, 100 nM DEX, 0.5 µM ATRA, 10 nM E2 + 100 nM DEX, or 10 nM E2 + 100 nM DEX + 0.5 µM ATRA. Ethanol was used as control for the DEX, DMSO for ATRA, and ethanol + DMSO as control for the combination treatments. Representative phase contrast microscopy images from three independent experiments. Scale bars indicate 20 µm.

B) Quantification of neurites from the experiment in **A**. Data is represented as the number of neurites per cell in three different images from three independent experiments. The brackets in the graph represent significance of all the experimental conditions *versus* their

controls. Statistical analysis: *t*-test, with * and *** indicating $p < 0.05$, and $p < 0.001$, respectively.

C) Western blot of the indicated proteins in BE(2)-GR+EV and BE(2)-GR+ERα cells following treatment with 10 nM E2, 100 nM DEX, 0.5 μM ATRA, or their combination during seven days. Proteins were separated on two gels with β-actin as loading control. Molecular weight markers shown to the left. Representative blots from three independent experiments.

D) Densiometric analysis of to Western blots from three independent experiments of the indicated proteins from BE(2)-GR+EV and BE(2)-GR+ERα presented in **C** (MYCN) and in **Figure 3B**. Data is shown as mean ± SD of two ($p75^{NTR}$) or three independent experiments; statistical analysis: *t*-test, with *, **, and **** indicating $p < 0.05$, $p < 0.01$, and $p < 0.0001$.

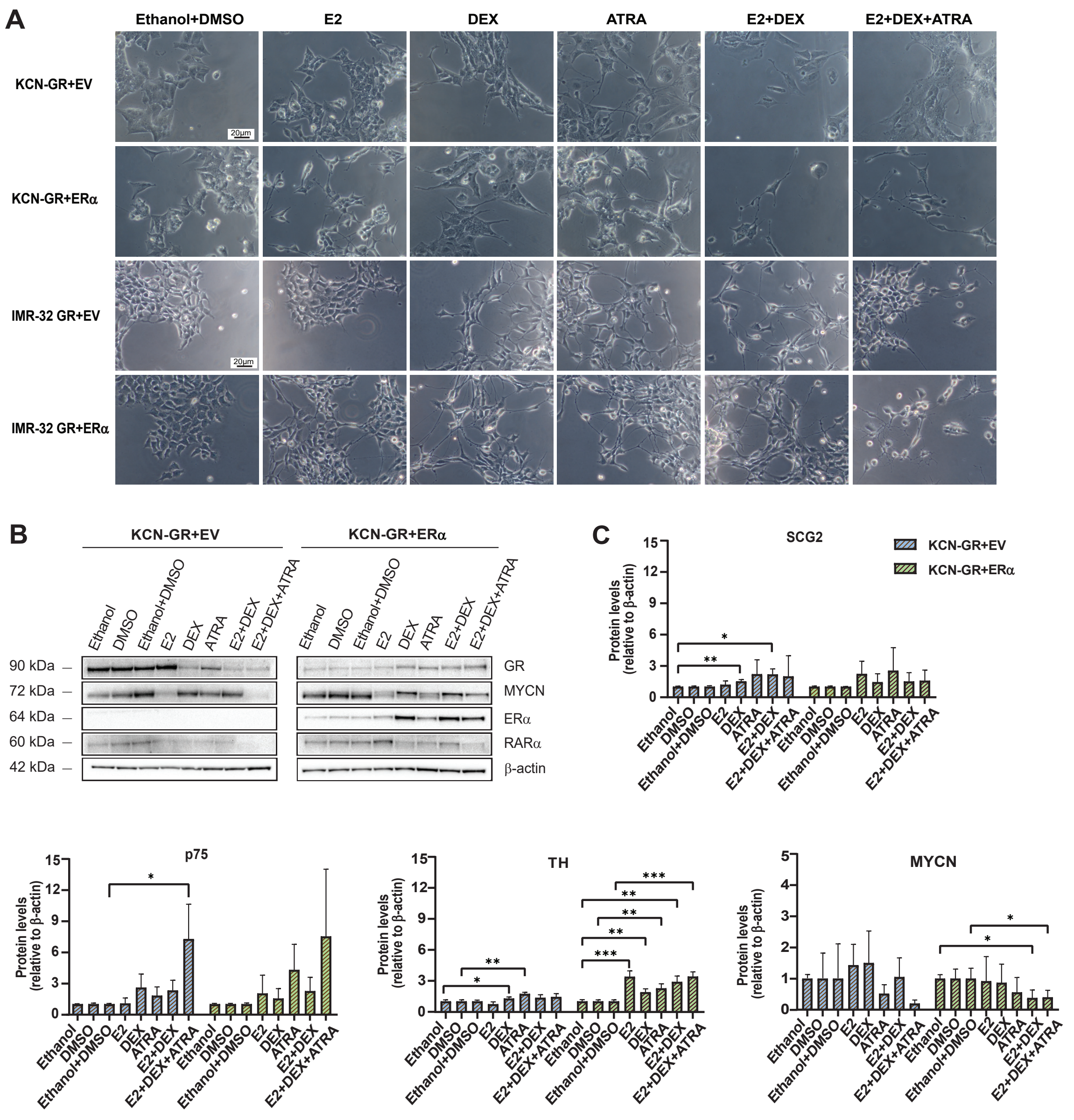

Supplementary Figure 6 related to Figure 4.

A) Neurite outgrowth assay after seven days of treatment with 20 nM of E2, 200 nM DEX, 1 μM of ATRA, 20 nM E2 + 200 nM DEX, or 20 nM E2 + 200 nM DEX + 1 μM of ATRA for KCN-GR+EV and KCN-GR+ERα cells, and for IMR32-GR+EV and IMR32-GR+ERα cells. Ethanol was used as control for DEX, DMSO for ATRA, and ethanol + DMSO as control for the combination treatments. Representative phase contrast microscopy images from three independent experiments. Scale bars indicate 20 μm.

B) Western blot of the indicated proteins in KCN-GR+EV and KCN-GR+ERα cells following treatment with 20 nM E2, 200 nM DEX, 1 μM ATRA, or their combination

during seven days. β-actin was used as loading control. Molecular weight markers shown to the left. Representative blots from three independent experiments.

C) Densiometric analysis of the Western blots from three independent experiments of the indicated proteins from KCN-GR+EV and KCN-GR+ERα cells represented in **B** (MYCN) and in **Figure 4B**. Data is shown as mean ± SD of three independent experiments; statistical analysis: *t*-test, with *, **, and *** indicating $p < 0.05$, $p < 0.01$, and $p < 0.001$.

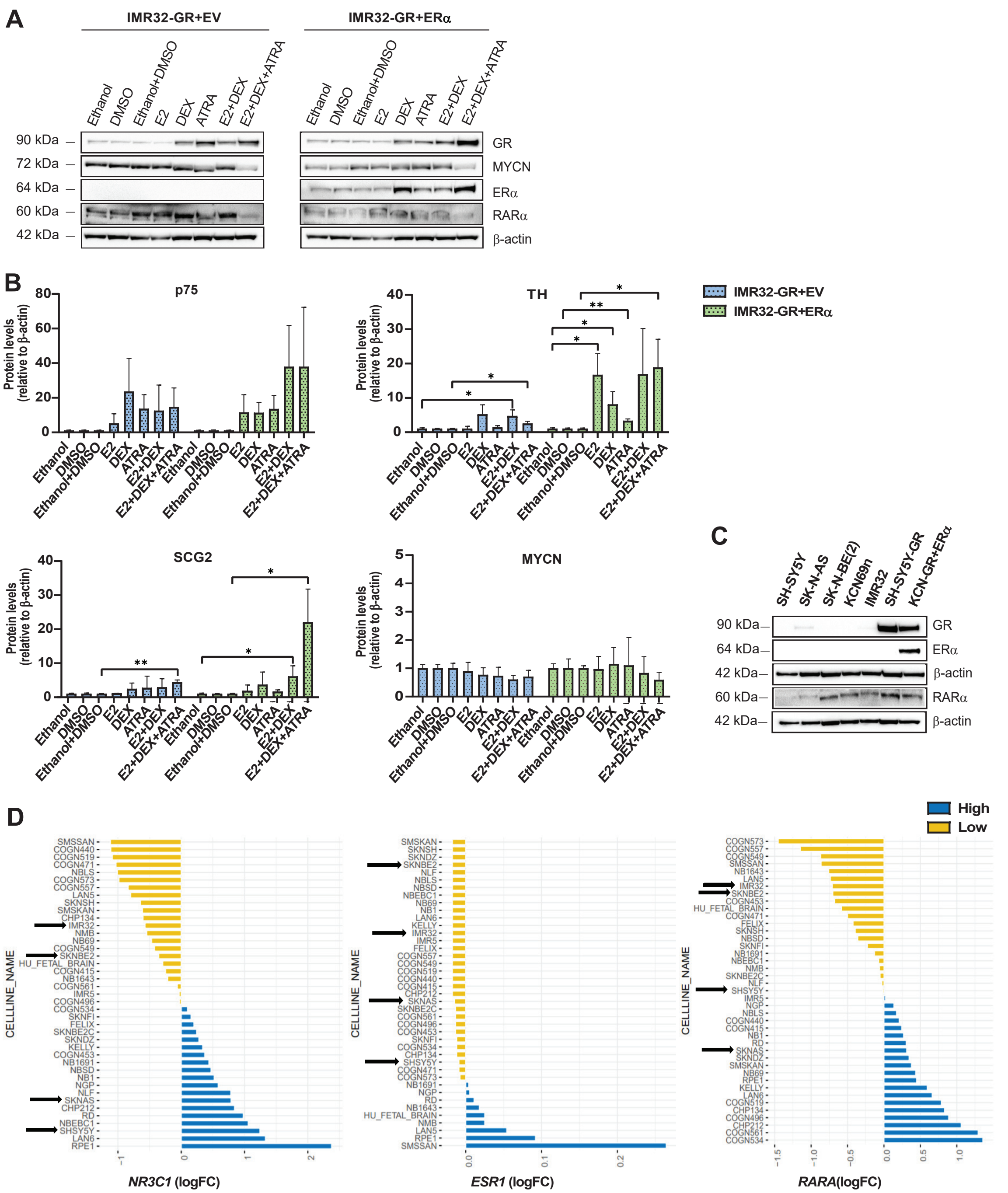

Supplementary Figure 7 related to Figure 4.

A) Western blot of the indicated proteins in IMR32-GR+EV and IMR32-GR+ERα cells treated with 20 nM E2, 200 nM DEX, 1 μM ATRA, or their combination during seven days. β-actin was used as loading control. Molecular weight markers shown to the left. Representative blots from three independent experiments.

B) Densiometric analysis corresponding to Western blots from three independent experiments of the indicated proteins from IMR32-GR+EV and IMR32-GR+ERα cells represented in **A** (MYCN) and in **Figure 4B**. Data is shown as mean ± SD of three independent experiments; statistical analysis: *t*-test, with *, and **, indicating $p < 0.05$, $p < 0.01$.
C) Western blot of the indicated proteins in parental SH-SY5Y, SK-N-AS, SK-N-BE(2), KCN-69n, and IMR32 cells. SH-SY5Y-GR and KCN-GR+ERα cells were used as positive controls for GR and ERα, respectively. Proteins were separated on two gels with β-actin as loading control. Molecular weight markers shown to the left. Representative blots from three independent experiments.
D) Analysis of the levels of *GR* (*NR3C1*), *ERα* (*ESR1*), and *RARα* (*RARA*) in 39 commonly used NB cell lines. The expression is presented as log (fold change). Arrows point to the cell lines used in this study.

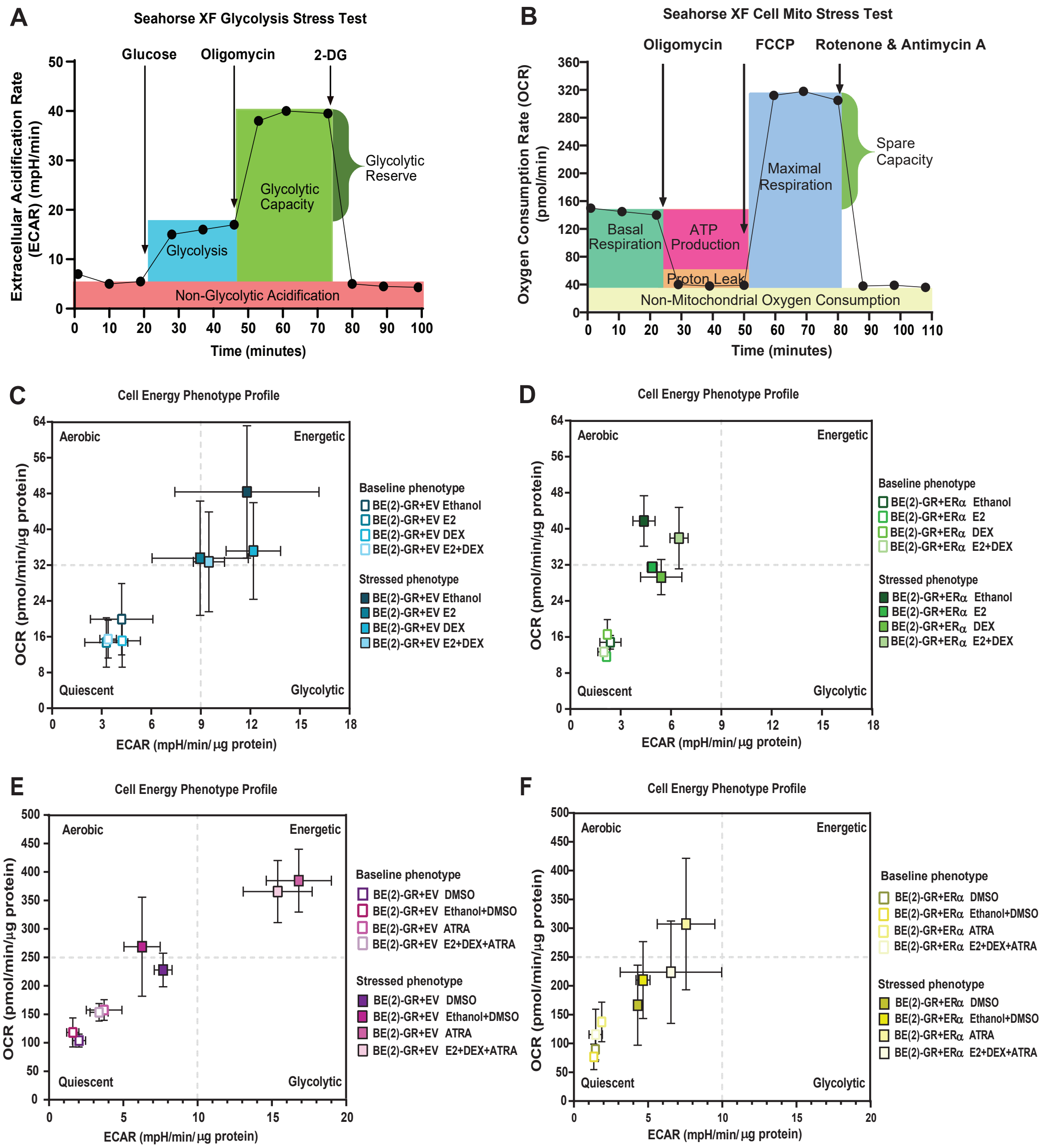

Supplementary Figure 8 related to Figure 5.

A) Graphical presentation of the principle of the Glycolysis Stress test. This assay measures the Extracellular Acidification Rate (ECAR), providing a method to analyze key glycolytic parameters: glycolysis, glycolytic capacity, glycolytic reserve, and non-glycolytic acidification. Glycolysis is defined by the ECAR rate reached after adding glucose. The maximum ECAR rate values after adding the ATP synthase inhibitor, Oligomycin, defines the glycolytic capacity. The glycolytic reserve is the ability to respond to an energy demand and it is the difference between the glycolytic reserve and glycolysis. By adding 2-deoxy-

glucose (2-DG), it is confirmed that ECAR produced in the experiment is due to glycolysis. The non-glycolytic acidification is the ECAR produced by other sources than glycolysis.
B) Graphical presentation of the principle of Mito stress test. This assy is used to assess the mitochondrial function by measuring the oxygen consumption rate (OCR), analyzing several mitochondrial parameters: basal respiration, ATP production, proton leak, maximal respiration, spare respiratory capacity, and non-mitochondrial respiration. The initial OCR value represent basal cellular respiration. The decrease in OCR after injecting oligomycin correlates to the basal respiration used to ATP production. Proton leak is calculated by the remaining basal respiration not linked to ATP production and that could indicate mitochondrial damage. The maximal OCR reached after injecting the uncoupler agent carbonyl cyanide-4 (trifluoromethoxy) phenylhydrazone (FCCP), and that produces the collapse of the proton gradient represents the maximal respiration. The spared respiratory capacity is the difference between maximal and basal respiration, indicating the flexibility of a cell to adapt to energy demand. The OCR value after addition of rotenone and antimycin A is considered as non-mitochondrial respiration.
C-D) Baseline and stressed metabolic phenotypes (indicated as open and filled squares, respectively) of **C)** BE(2)-GR+EV and **D)** BE(2)-GR+ERα cells after treatment during 72 h with ethanol, 10 nM E2, 100 nM DEX, or the combination of 10 nM E2 + 100 nM DEX. Quantification is presented in **Figure 5E** and **Figure 5G**.
E-F) Baseline and stressed metabolic phenotype (indicated as open and filled squares, respectively) of **E)** BE(2)-GR+EV and **F)** BE(2)-GR+ERα cells after treatment during 72 h with DMSO, ethanol, 0.5 μM ATRA, or the triple combination of 10 nM E2 + 100 nM DEX + 0.5 μM ATRA. Quantification is presented in **Figure 5F** and **Figure 5H**. All experiments in **C-F** were carried out three independent times.
Images in **A-B** are adapted with permission from Seahorse Bioscience, Agilent Technologies, North Billerica, MA, USA.

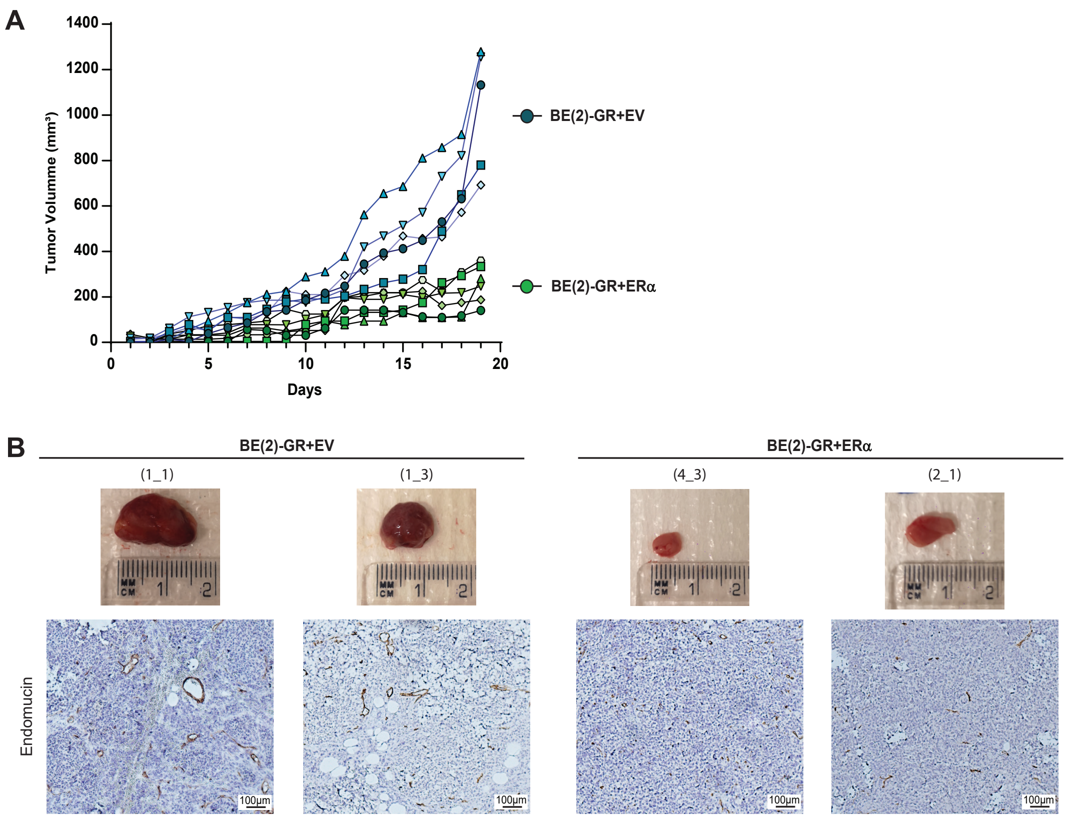

Supplementary Figure 9 related to Figure 6.

A) Tumor volume for each mouse in the xenograft experiment comparing tumors generated from BE(2)-GR+EV (blue) and BE(2)-GR+ERα (green) cells, summarized in **Figure 4A**; *n*= 5 in the BE(2)-GR+EV and *n*= 6 in the BE(2)-GR+ERα groups, respectively. Tumor growth was followed until the control group reached the ethical endpoint volume of 1 cm^3. **B)** Microscopic images of immunohistochemistry analysis of two BE(2)-GR+EV and two BE(2)-GR+ERα xenograft tumors stained with anti-endomucin. Scale bars indicate 100 µm. Pictures are representative of at least five independent stainings per condition.

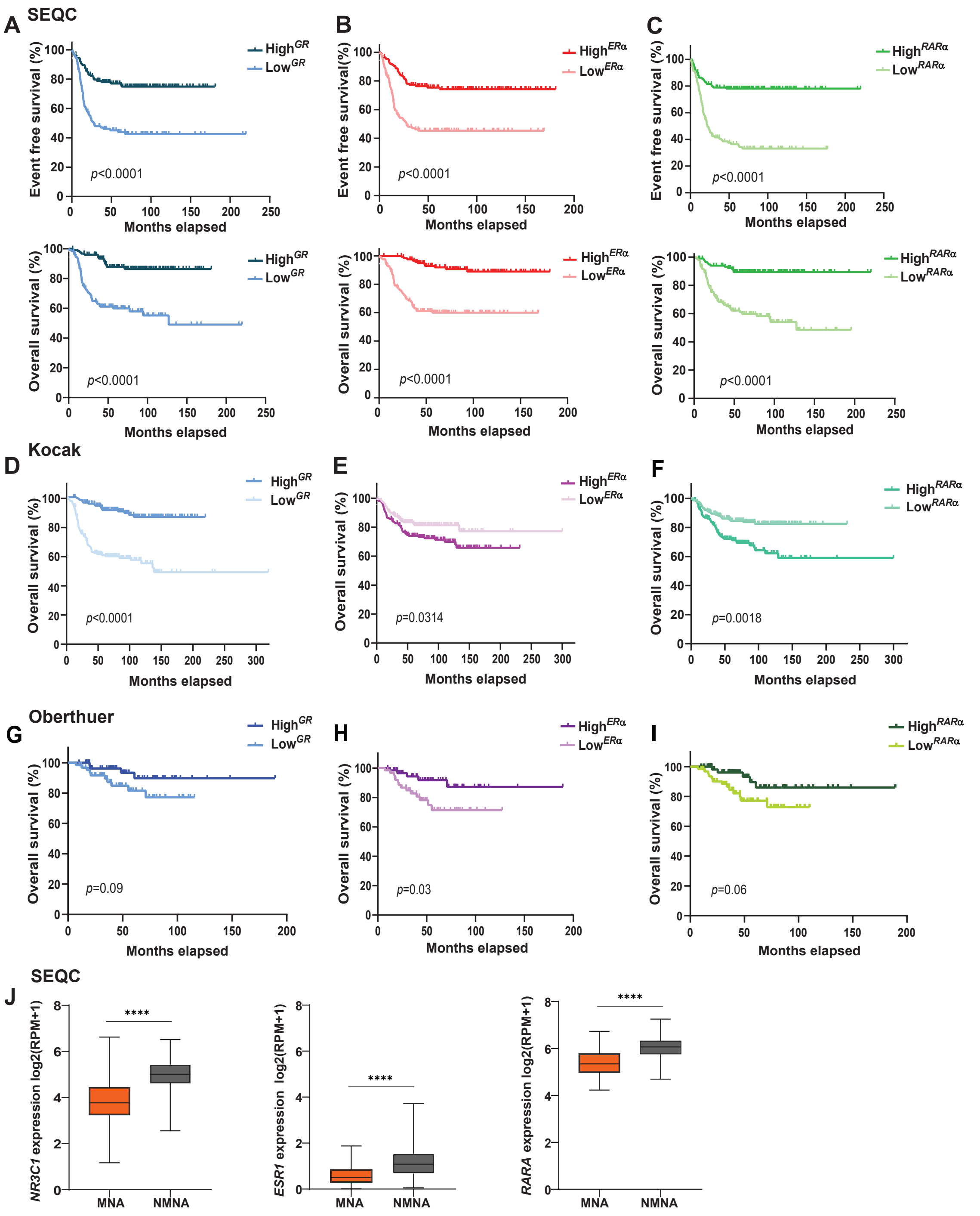

Supplementary Figure 10 related to Figure 7.

A-C) Kaplan-Meier overall and event free survival curves of patients from the SEQC NB dataset, divided in two groups according to high or low *GR*, *ERα,* or *RARα* mRNA expression levels: High^{GR} *versus* Low^{GR}, High$^{ER\alpha}$ *versus* Low$^{ER\alpha}$, and High$^{RAR\alpha}$ *versus* Low$^{RAR\alpha}$. Log-rank test was used for analysis. *P* values are shown in the plots.

D-F) Kaplan-Meier overall survival curves of patients from the Kocak NB dataset, divided in two groups according to high or low *GR*, *ERα,* or *RARα* mRNA expression levels: $High^{GR}$ *versus* Low^{GR}, $High^{ERα}$ *versus* $Low^{ERα}$, and $High^{RARα}$ *versus* $Low^{RARα}$. Log-rank test was used for analysis. *P* values are shown in the plots.
G-I) Kaplan-Meier overall survival curves of patients from the Oberthuer NB dataset, divided in two groups according to high or low *GR*, *ERα,* or *RARα* mRNA expression levels: $High^{GR}$ *versus* Low^{GR}, $High^{ERα}$ *versus* $Low^{ERα}$, and $High^{RARα}$ *versus* $Low^{RARα}$. Log-rank test was used for analysis. *P* values are shown in the plots.
J) mRNA expression of the *GR* (*NR3C1*), *ERα* (*ESR1*), and *RARα* (*RARA*) genes in the *MYCN*-amplified (MNA; *n*= 92) and non-*MYCN*-amplified (NMA; *n*= 401) group of patients from the SEQC dataset. As the *MYCN* status was unknown for five patients, the analysis was carried out on 493 patients. Statistical analysis: *t*-test with ns, **, ***, and **** indicating non-significant, $p < 0.01$, $p < 0.001$, and $p < 0.0001$, respectively.

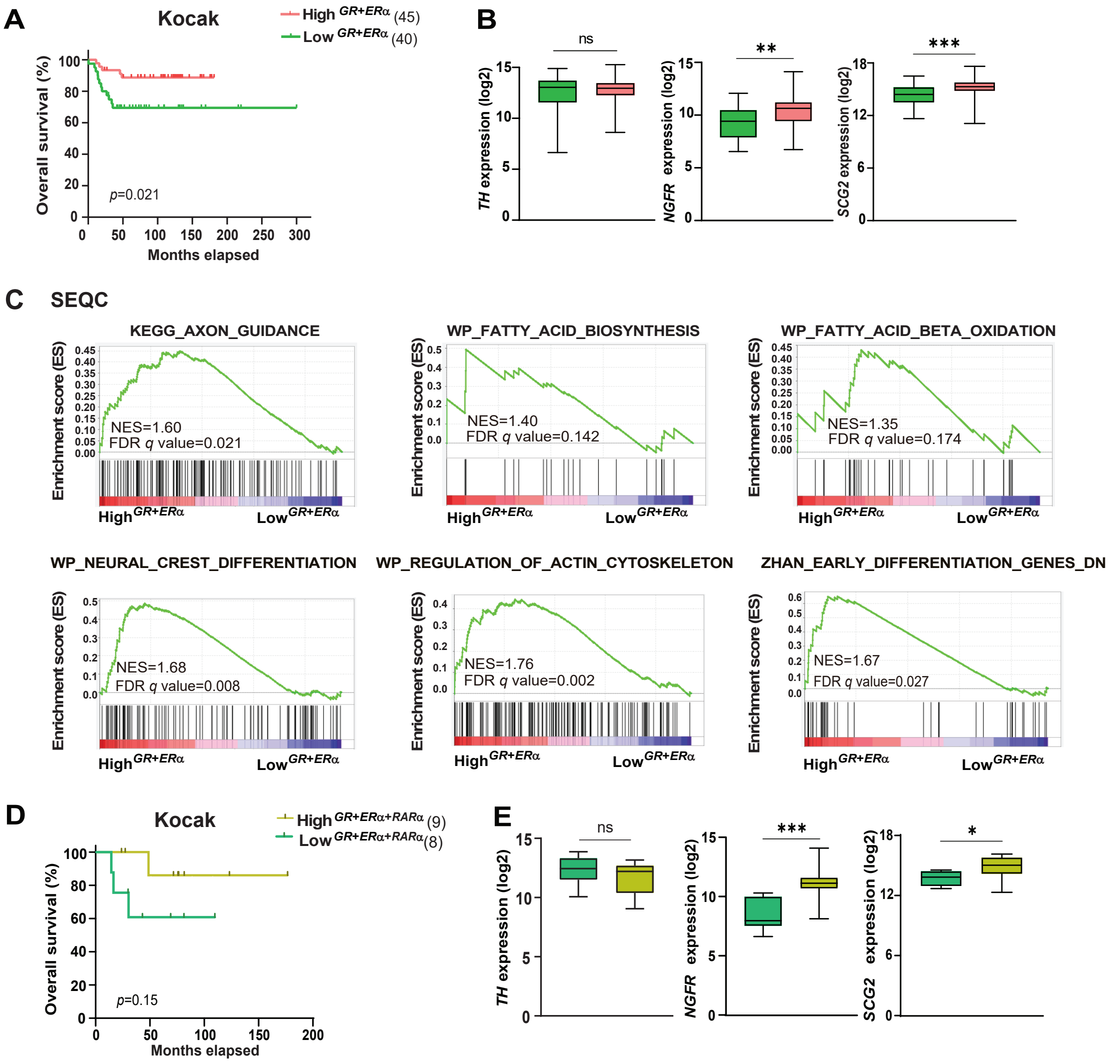

Supplementary Figure 11 related to figure 7.

A) Kaplan-Meier overall survival curve of NB patients from the Kocak dataset, divided into two groups according to combined *GR* and *ERα* mRNA expression levels: High$^{GR+ERα}$ (45 patients) *versus* Low$^{GR+ERα}$ (40 patients).

B) mRNA expression of the neuronal differentiation markers *TH*, *NGFR*, and *SCG2* in the High$^{GR+ERα}$ *versus* Low$^{GR+ERα}$ group of patients from the Kocak dataset. Statistical analysis: *t*-test with ns, **, and ***, indicating non-significant, $p < 0.05$, $p < 0.01$, $p < 0.001$, respectively.

C) Gene set enrichment plots of differentiation and metabolic related processes comparing genes expressed in patients with combined high *versus* low *GR* and *ERα* mRNA expression levels (High$^{GR+ERα}$ *versus* Low$^{GR+ERα}$) from the SEQC cohort. Plots were obtained from GSEA (v4.0.3) using the C2 curate set collection presented in **Additional File 1**. FDR *q*-

value < 0.05 was considered significantly enriched. Normalized enrichment score (NES) was defined as actual ES/mean (ES against all permutations of the dataset).

D) Kaplan-Meier overall survival curve of patients from the Kocak cohort, divided into two groups according to triple combined *GR*, *ERα,* and *RARα* mRNA expression levels: High$^{GR+ER\alpha+RAR\alpha}$ (nine patients) *versus* Low$^{GR+ ER\alpha+RAR\alpha}$ (eight patients).

E) Plots of mRNA expression of the neuronal differentiation markers *TH*, *NGFR*, and *SCG2* between the High$^{GR+ ER\alpha+RAR\alpha}$ *versus* Low$^{GR+ ER\alpha+RAR\alpha}$ groups of patients from the Kocak dataset. Statistical analysis: *t*-test with ns, *, and ***, indicating non-significant, $p < 0.05$, $p < 0.001$, respectively.

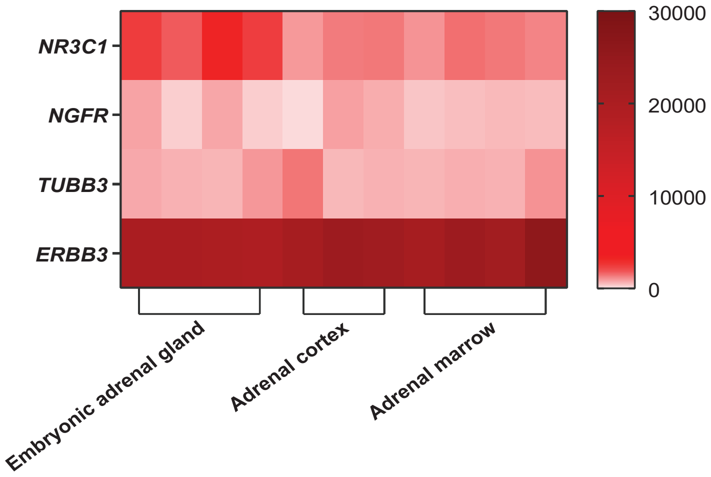

Supplementary Figure 12 related to Figure 8.

Heatmap representing expression of *NR3C1* (*GR*) and the neural differentiation markers *NGFR* (*p75*^{NTR}) and *TUBB3* (*βIII-tubulin*), as well as the neural progenitor marker *ERBB3* in human tissue from four embryonic adrenal glands, three adult adrenal cortex tissues, and four adrenal marrows as indicated. Expression scores were obtained from the Suntsova dataset. The expression score of each gene is shown in the color bar shown to the right, light red for lower towards dark red for higher expression score.

Additional file 1. Gene set enrichment analysis of patients with High$^{GR+ER\alpha}$ *versus* Low$^{GR+ER\alpha}$ mRNA expression from the SEQC NB dataset (C2 curate set collection). Related to **Supplementary Figure 11**.

Additional file 2. Gene set enrichment analysis of patients with High$^{GR+ER\alpha}$ *versus* Low$^{GR+ER\alpha}$ mRNA expression from the SEQC NB dataset (C5 ontology set collection). Related to **Supplementary Figure 11**.
